# The colonization and divergence patterns of Brandt’s vole (*Lasiopodomys brandtii*) populations reveal evidence of genetic surfing

**DOI:** 10.1186/s12862-017-0995-y

**Published:** 2017-06-21

**Authors:** Ke Li, Michael H. Kohn, Songmei Zhang, Xinrong Wan, Dazhao Shi, Deng Wang

**Affiliations:** 1 College of Plant Protection, China Agricultural University, 2 Yuanmingyuan West Road, Haidian District, Beijing, 100193 China; 2 Institute of Biosciences and Bioengineering, Rice University, 130 Anderson Biology, P.O. Box 1892, Houston, 77251-1892 USA; 3 State Key Laboratory for Integrated Management of Pest Insects and Rodents, Institute of Zoology, Chinese Academy of Sciences, 1 Beichen West Road, Chaoyang District, Beijing, 100101 China

**Keywords:** *Lasiopodomys brandtii*, Ancestral area, Migration, Range expansion, Genetic surfing

## Abstract

**Background:**

The colonial habit of Brandt’s vole (*Lasiopodomys brandtii*) differs from that of most other species of the genus *Microtus*. The demographic history of this species and the patterns shaping its current genetic structure remain unknown. Here, we explored patterns of genetic differentiation and infered the demographic history of Brandt’s vole populations through analyses of nuclear microsatellite and D-loop sequences.

**Results:**

Phylogenetic analyses divided the sampled populations into three main clusters, which represent the southeastern, northeastern and western parts of the total range in Mongolia and China. Molecular data revealed an ancestral area located in the southeast of the extant range, in the Xilinguole District, Inner Mongolia, China, from where Brandt’s vole populations began expanding. A gene flow analysis suggested that the most likely colonization route was from the ancestral area and was followed by subsequent northeastward and westward range expansions. We identified decreases in genetic diversity with increasing distance from the founder population within the newly occupied regions (northeastern and western regions), clinal patterns in the allele frequencies, alleles that were rare in the original area that have become common in the newly occupied regions, and higher genetic differentiation in the expanded range compared with the original one.

**Conclusion:**

Our results indicate that *L. brandtii* most likely originated from the southeastern part of its current geographic range, and subsequently colonized into the northeastern and western parts by expansion. The genetic patterns among the derived populations and with respect to the original population are consistent with that expected under genetic surfing models, which indicated that genetic drift, rather than gene flow, is the predominant factor underlying the genetic structure of expanding Brandt’s vole populations.

**Electronic supplementary material:**

The online version of this article (doi:10.1186/s12862-017-0995-y) contains supplementary material, which is available to authorized users.

## Background

The evolutionary history of most species generally is characterized by an interplay among environmental changes, episodes of range expansion and migration, population admixtures, and local extinctions [1, 2]. These and other factors that play roles in the past leave detectable signatures in the genetic structures of modern populations [3]. For a number of Arvicolinae rodents in the Northern Hemisphere such population genetic and phylogeographic studies have been reported [3–8].

Brandt’s vole (*Lasiopodomys brandtii*) (Radde, 1861) is a steppe-dwelling rodent species that is currently distributed from the central parts of Inner Mongolia, through the central and eastern of Republic of Mongolia, and to the southern borders of Mongolia in Trans-Baikalia, Russia [9, 10]. Brandt’s vole forms its own lineage among arvicolids rodents [10]. Over the past decades, the evolutionary origins of Brandt’s vole have been studied based on paleontological, cytological and nuclear DNA data [10–15]. However, prior to this study, no direct molecular evidence ragarding the geographical origins of the ancestral populations or their colonization and differentiation patterns has been reported.

Brandt’s vole is well adapted to the colonization of patchy, ephemeral habitats [16, 17], where the populations exhibit seasonal and multi-annual fluctuations in abundance [17–19]. The species distributed discontinuously across the Mongolian plateau [20]. However, the habitats are rather homogeneous [20] with respect to plant community attributes, particularly species composition, vegetation height and degree of plant cover [21]. Brandt’s vole prefers degraded grasslands [22], which are often interspersed with less favorable habitats characterized by livestock grazing [16, 21, 23]. The populations of Brandt’s vole show declines when the grass is shorter than approximately 5 cm and sparse (less than 40% cover), and do not persist well in habitats with dense coverage (more than 80%) by tall grasses (more than 17 to 20 cm) [17, 21, 24]. The environmental attributes affecting Brandt’s vole populations generally are related to climate and human activities [25, 26].

Climatic changes that alter the availability of suitable habitat have can trigger range shifts and local extinctions of populations for most organisms [27]. Such effects have also been observed on the Mongolian plateau, where Brandt’s vole occurs. There cold and dry climate during the Pleistocenens led to increased coverage by tundra steppe and then dry steppe habitats [28, 29]. The forest-steppe boundary of the plateau was situated further south during the early-mid Holocene stage (10–5 kyr BP), which caused by the enhanced aridity occurred during the period [25]. Currently, it is believed that climate change and overgrazing are driving major factors that result in the degradation and desertification of Mongolian plateau pastureland [30, 31]. The shifts in the distribution of Brandt’s vole populations are expected to result from species migration and/or adaptation to the environment. Biotope evolutionary changes, such as soil development [26], hydrological changes [32], desertification [33], and landscape evolution [34] in the Mongolia plateau inhabited by extant and extinct Brandt’s voles should impact the population dynamics. Such changes should also interfere with the genetic composition of the population. Through fine-scale geographic sampling in well-defined ecological and historical contexts, it is possible to detect cases of spatially varying selection that involve subtle shifts in the allele frequency among locally adapted populations [35–38].

Here we used microsatellite data and mtDNA D-loop sequences to describe the phylogeography and genetic diversity of Brandt’s vole populations over space and time, and to infer the population demography history. We reconstructed the likely geographic origin of the species and past colonization routes. Genetic surfing is a phenomenon associated with species displaying rapid population growth, a patchy distribution, and rapid population growth [27, 39]. As part of the genetic surfing phenomenon, the frequency of alleles arising on the leading edges of the wave of range expansion can increase to high levels due to genetic drift; in other words, once rare alleles might become predominant in populations colonizing the new territory [27]. The phenomenon has attained some attention because under such a scenario, the relative position of an individual and the alleles it carries more strongly affect the fate of alleles than selection or standard genetic drift occurring in a Fisher-Wright population or stable populations of small effective size [39]. Thus, during our study we paid particular attention to the possibility of empirically demonstrating the phenomenon in Brandt’s vole.

## Methods

### Sampling

**Fig. 1:**
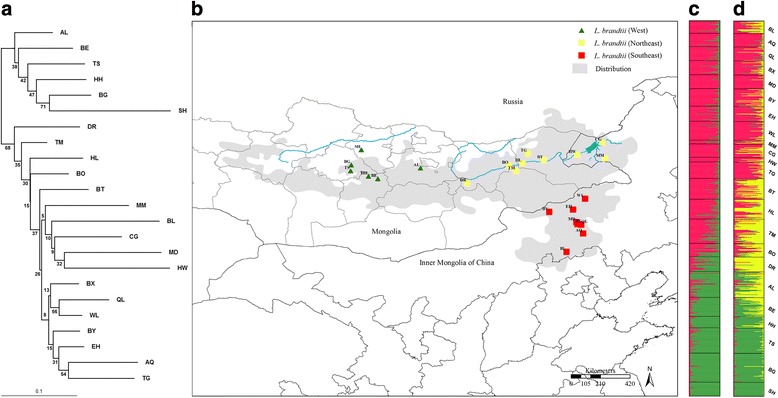
Population structure of Brandt’s vole (*N* = 814) from 23 sites based on nuclear microsatellite data. **a** Neighbor-joining (NJ) tree based on pairwise genetic distances (D_a_) between sampling sites depicted in **b**); **c** Bayesian structure plot for K = 2. **d** Analysis with K = 3. Coloring scheme of symbols reflects STRUCTURE results. Detailed sampling information is provided in Additional file 1: Table S1

A total of 851 Brandt’s voles were captured using live-traps from 23 sites in Inner Mongolia (China) and Mongolia (Fig. 1b). Twelve populations from Mongolia were collected in July 2010, whereas the populations from Inner Mongolia were trapped in July 2008 and 2009. A summary of the populations is provided in Additional file 1: Table S1. All samples were captured by regular live trapping for two successive days at geographic locations spanning >5 hectars (ha) in size. Traps were baited with peanuts and set at burrow entrances in the morning until no new voles entranced. Each captured animal was weighed, sexed and was given unique identification numbers by toe-clipping according to Wood & Slade [40]. Adults were selected for the analyses to avoid obvious sampling parent-offspring pairs. All toe clippings were immediately placed in 70% ethanol and stored at −20 °C until used for DNA extraction. A total of 26–63 individuals were collected from each site with the exception of Hangwula and Modamuji, where trapping success was low.

### DNA extraction, microsatellite genotyping and D-loop sequencing

Genomic DNA was extracted using the proteinase-K digestion and phenol-chloroform extraction method as described by Sambrook et al. [41]. All individuals were genotyped at 12 microsatellite loci [42, 43]. Primer sets are provided in Additional file 1: Table S2. DNA amplification was performed in a thermocycler (Model 9700, Applied Biosystems, USA). The 15-μL reaction mixtures contained ~50 ng of DNA, 1× PCR buffer, 0.2 μM of each primer, 1.0–2.5 mM MgCl_2_, 0.2 mM of each nucleotide, 1 U of Taq polymerase (TaKaRa Bio Company, Dalian, China), and ddH_2_O. The amplification program consisted of an initial denaturation for 10 min at 95 °C followed by 35 cycles of 40 s at 94 °C for denaturation, 40 s for annealing, and 40 s at 72 °C for extension and a final extension for 10 min at 72 °C. The PCR products were analyzed using an ABI PRISM3730 DNA Sequencer (Applied Biosystems). Genotypes were scored using GeneMarker 1.7 (Applied Biosystems). Two observers identified genotypes independently. If the genotypes of the same vole and locus differed, the genotyping process was repeated until a consensus was reached. Allelic dropout and the presence of null alleles were screened using Micro-Checker version 2.2 [44]. Ninety-eight randomly selected samples (12% of all samples) were genotyped again, and the results were analyzed using the Gimlet program [45] to estimate the rates of different types of errors.

A 734-bp fragment of the D-loop sequence was amplified by PCR from 746 individuals using the primer pairs (5′-ACCATCAACACCCAAAGC-3′ and 3′-GTACTTGATACCCTCTCC-5′) designed with Primer 5 [46] based on the sequence of *Lasiopodomys mandarinus* with GenBank accession number NC_025283.1. The PCR was performed in 25-μL reaction mixtures containing ~25 ng of genomic DNA, 1× PCR buffer, 0.2 μM of each primer, 1.5 mM MgCl_2_, 0.2 mM of each nucleotide, 1 U of Taq polymerase (TaKaRa Bio Company, Dalian, China), and ddH_2_O. The reaction was optimized and programmed over 35 cycles using a GeneAmp PCR system 9700 thermocycler (Applied Biosystems) with the following temperature profile: 5 min at 94 °C for denaturation; followed by 10 cycles of 30 s at 94 °C for denaturation, 30 s at 60 °C for annealing, and 70 s at 72 °C for extension; 15 cycles of 30 s at 94 °C, 30 s at 55 °C, 70 s at 72 °C; and 10 cycles of 94 °C for 30 s, 50 °C for 30 s, 72 °C for 70 s; and a final extension step for 7 min at 72 °C. All PCR products were sequenced in a single direction using an automated ABI 3730 DNA sequencer (Sangon Biotech Ltd). The sequences were aligned to the corresponding D-loop regions of *L. mandarinus* using the programs Lasergene 7.2 [47] and CLUSTALX [48] and were checked and edited manually using Lasergene 7.2. The D-loop haplotype sequences from Brandt’s voles were deposited in GenBank (GenBank nos: KY354521-KY354550).

### Genetic diversity and phylogenetic analyses

Hardy-Weinberg equilibrium (HWE) tests based on the excess of heterozygosity were performed for all loci using GENEPOP v4.5 [49]. Each test was run for 1000 dememorization steps, followed by 100 batches of 1000 steps each. *P*-values were adjusted using the Bonferroni correction [50]. We used GENALEX 6.5 [51] to estimate the number of alleles (*N*_*a*_), allele frequencies, private and rare alleles (allelic frequencies under 0.05), and observed (*H*_*o*_) and expected heterozygosity (*H*_*e*_) for each sampling site and for cluster inferred from phylogenetic analyses (see below). Pairwise population differences were also explored with the use of Wright’s *F*_ST_ values. The allelic richness (*A*_*r*_) was calculated for each site population based on a minimum sample size of 14 using the rarefaction procedure implemented in HP-Rare [52].

We used Poptree2 [53] to construct a neighbor-joining (NJ) tree for all Brandt’s vole populations based on the pairwise genetic distances (D_a_) generated from microsatellite genotypes, and we used a Bayesian MCMC assignment method implemented in STRUCTURE 2.3 [54] to assign individuals to inferred clusters based on multilocus genotypes. The admixture ancestry and the correlated allele frequencies model were implemented in STRUCTURE, with K (the number of clusters) set to a value between 1 and 23, and 20 independent iterations consisting of 100,000 burn-in steps and an iteration length of 100,000 were performed for each value of K. The results subsequently were processed by STRUCTURE HARVESTER (http://taylor0.biology.ucla.edu/structureHarvester/) [55] to delineate the most likely level of population subdivision (the appropriate K), which was identified using the maximal values of a statistic ΔK based on the rate of change in the log probability of the data between successive K-values [56]. The output from STRUCTURE was visualized using the DISTRUCT program (http://www.stanford.edu/group/rosenberglab/distructDownload.html). We also performed AMOVA analysis with ARLEQUIN [57] to test for the genetic differentiation within and among clusers.

**Fig. 2:**
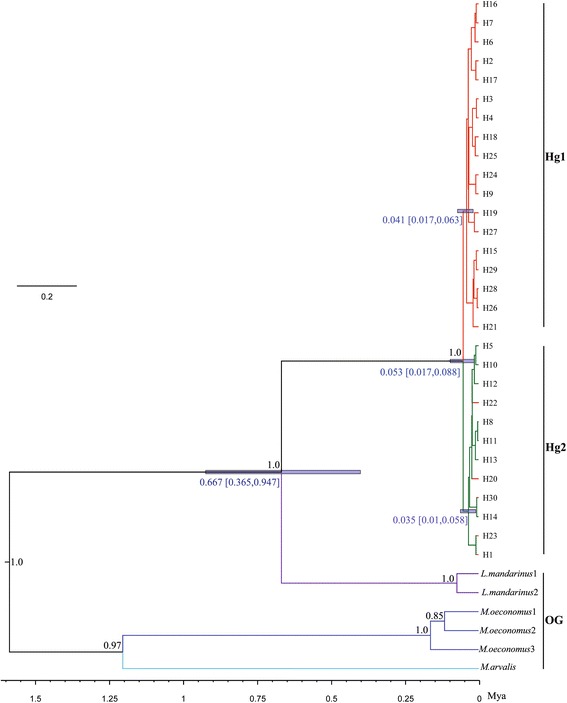
Phylogenetic analysis of Brandt’s vole. Maximum clade credibility tree reconstructed using D-loop haplotypes. The posterior probability (≥0.85) clades are presented at nodes (*black numbers*). *Blue bars* correspond to the 95% HPD for TMRCA (*blue numbers*). *L. mandarinus*, *M. oeconomus* and *M. arvalis* are used as outgroups. *Branch color* denotes the geographic distribution of haplotypes (coloring scheme as in Fig. 3c)

**Fig. 3:**
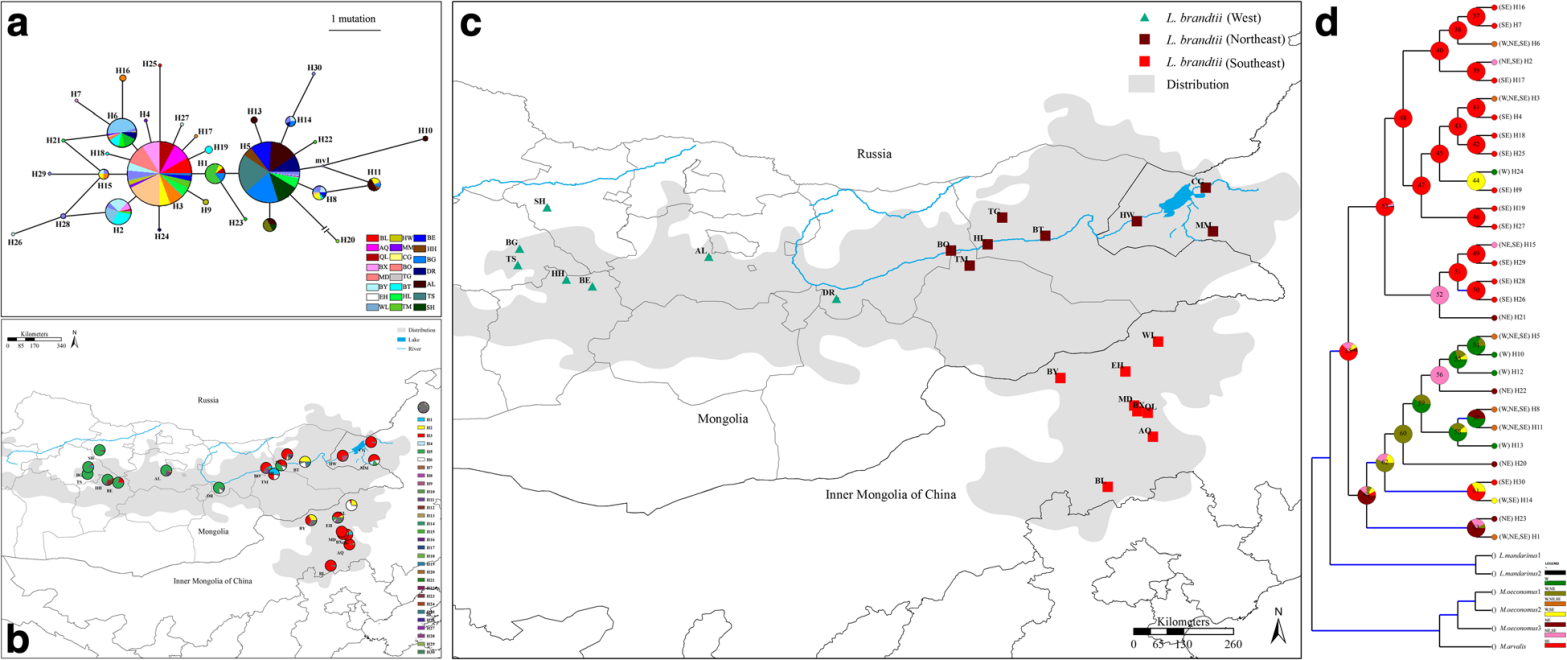
Population structure and history of Brandt’s vole (*N* = 746) from 23 sites inferred from D-loop data. **a** Median-joining network of 30 haplotypes. *Circle sizes* indicate haplotype frequencies. The colored segments indicate the sample size of voles available for each geographic location specified by the color key (*black square*, mv1, represents missing or unsampled haplotypes. **b** Geographic distributions of haplotypes found in **c**. **d** Ancestral area reconstruction, where W, NE and SE represent western, northeastern and southeastern distributions, respectively (color coding as in **c**). Pie charts on each node show the posterior probability (PP) of each ancestral haplotype occurring at an inferred ancestral geographic location as inferred with the S-DIVA method. Node codes (37–65) are shown on pie charts. (c.f. Additional file 3: Table S9 for numerical results obtained using S-DIVA and DEC). Probabilities <5% were lumped together as “*”. *Blue branches* indicate *PP* > 0.69

Polymorphism indices of the D-loop sequences including the number of haplotypes (N), nucleotide diversity (π) and haplotype diversity (*Hd*), were calculated for each sampling site, each cluster and diverged groups of haplotypes (haplogroups obtained from phylogenetic analysis, c.f. Figs. 2 and 3a) using DNASP 5.0 [58].

We estimated the phylogenetic relationships among haplotypes using the Bayesian method implemented in BEAST v2.1.3 [59]. We consider three rodent species to be the most adequate outgroups of *L. brandtii*: *L. mandarinus* (GenBank nos. NC_025283.1, JX014233.1 and KF819832.1) always form the sister group with *L. brandtii*, and it can be rather closely related but clearly outside of *L. brandtii* in the phylogenetic relationship [3, 60]. *Microtus oeconomus* (GenBank nos. HM135921, AJ616853 and HM135928) and *Microtus arvalis* (GenBank no. KP013595) have more distant in the molecular phylogeny of *Microtus* species [3, 60, 61]. Paleontological and molecular phylogenetic analysis suggested that *Lasiopodomys* might be part of the sister clade to *Microtus* [10, 62]. We used the HKY substitution model as selected by Akaike’s Information Criterion [63] in MODELTEST [64] and a strict clock model generated by Bayes factors [65]. The Yule model was selected as tree prior model; MCMC chains were run for 50 million iterations with parameters sampled every 1000 iterations; Trees were combined in TreeAnnotator v2.1.3 [66] with 50% burn-in values. Tracer v1.5.0 [67] was used to check for the convergence of Markov chains and to ensure sufficient sampling. Expected sample sizes (ESSs) for all parameters were greater than 100. The maximum clade credibility tree was visualized using Figtree v1.4.2 [68]. We constructed a Median-Joining (MJ) network for D-loop haplotypes using Network5.0 [69]. We performed AMOVA to test for the differentiation within and among cluster and haplogroup using ARLEQUIN [57]. The geographic distributions of the haplotypes were visualized on a map using ArcGIS v10.1 [70].

Divergence times separating haplogroups were estimated using the Bayesian method implemented in BEAST v2.1.3. To conduct the dating runs, we manipulated a Calibrate Yule tree prior as the tree model and a strict clock model. The divergence time between *L.mandarinus* and *L.brandtii* (0.5–0.95 Ma) [3] was assumed to be the calibration time. MCMC chains were run for 50 million iterations with parameters sampled every 5000 generations and 10% burn-in values. Tracer 1.5 was used to assess ESSs for all paremeters and to verify that the posterior distribution of the divergence calibration matches the prior distribution. Trees were combined in TreeAnnotator v2.1.3 and node ages were visualized using Figtree v1.4.2.

### Inferring ancestral areas and modeling colonization routes

We used three clusters (as obtained from D-loop) for ancestral area reconstruction. They were partitioned in the western (W), southeastern (SE) and northeastern (NE) based on the D-loop phylogenetic analysis (Fig. 3c; Table 2). We performed a Statistical-Dispersal-Vicariance-Analysis (S-DIVA) in a Bayesian framework implemented in RASP v3.2 [71, 72], and Dispersal-Extinction-Cladogenesis process (DEC) in a likelihood framework using Lagrange [73, 74] to reconstruct the likely geographic history of Brandt’s vole. We ran S-DIVA on 10,000 trees from MCMC output to account for phylogeny uncertainties. We opted for not constraining the dispersal probabilities to avoid over-parameterization for the DEC analysis. The number of maximum areas was set as 2 for both two analyses. The input tree for the analyses was generated from BEAST (Fig. 2). For Lagrange, a Python script was created using the online Lagrange configurator.

To explore the dispersal process and historical gene flows, we applied MIGRATE-N v.3.6.1 [75, 76] to the microsatellite data for both two distinct clusters (eastern and western distribution) (Additional file 2: Fig. S1) and three clusters (southeastern, northeastern and western distribution) supported by the phylogenetic results (Fig. 1; Additional file 2: Fig. S1; Table 2). MIGRATE-N, implements coalescent-based MCMC simulations, was applied to estimate Θ, *M* and the marginal likelihood of the specified gene flow models [76]. The population size Θ is defined as 4*N*_*e*_μ, where *N*_*e*_ is the effective population size and μ is the mutation rate of loci per generation, and the migration rate *M* is equivalent to *m*/μ, where *m* is the immigration rate per generation [75]. Preliminary runs were performed using the full migration model to determine the optimal parameters. We performed runs through Bayesian inference using the following parameters: slice sampling, an exponential prior distribution for Θ (min: 0, mean: 35, max: 70), an exponential prior distribution for migration (min: 0, mean: 750, max: 1500), static heating with temperatures of 1.00, 1.50, 3.00 and 10^6^; potential occurrence of swapping among chains at every step; and 10^4^ burn-in steps followed 5 × 10^7^ parameter samplings recorded at intervals of 10^3^. We randomly selected 20 individuals per population for our MIGRATE-N analyses due to computational demands and the evidence that more than 20 individuals do not increase the accuracy of parameter estimations [77]. All final reported runs met the convergence criteria for ESSs greater than 10^4^ and showed good agreement between the mean and median estimates for all parameters. The posterior distribution plot of each parameter was also used to visually test for convergence. The marginal likelihood and probability of each model was approximated using a Bezier-corrected thermodynamic integration [76, 78].

### Testing for range expansion

We used DNASP 5.0 [58] to conduct neutrality tests [79–81] on D-loop sequence data. Past population size changes were inferred by computing a raggedness index (r) obtained from a site mismatch analysis [82, 83] and by comparing the observed distribution to the distribution under the null model of constant population size.

Effective population size fluctuations over time were inferred using a Bayesian Skyline plot method (BSP) implemented in the program package BEAST v2.1.3. The GTR model was run for the entire population and for each D-loop haplogroup separately. Substitution rate for the D-loop sequences estimated for *Meriones* was similar to *Microtus* [84]. We used the substitution rate of 1.27 × 10^−7^ substitutions per site per year in D-loop estimated for *Meriones meridianus* (95% confidence interval = 1.2 × 10^−7^ to 1.33 × 10^−7^/site/year) [84] to scale the time axis of BSPs. The chains were run for 200 (entire samples) and 40 (haplogroups) million iterations respectively, of which the first 10% were discarded. Model parameters were sampled every 1000 iterations. The posterior samples were combined using LogCombiner [59]. Skyline plots were generated using Tracer v1.5.0. ESSs for all parameters were greater than 100.

The isolation by distance was tested for all site populations in Brandt’s vole through a regression of pairwise *F*_ST_/ (1- *F*_ST_) values on the lnGD (geographic distance among populations) [85] using SPSS v.20 [86]. The pairwise *F*_ST_ valuas were calculated based on the microsatellite data using ARLEQUIN 3.5 [57]. We also performed IBD tests for different clusters as described above (Fig. 1b).

In a pairwise manner for each of the three clusters, we iteratively calculated the average *F*_ST_ between samples obtained from one site of each cluster and all samples from all sites that are part of another cluster. According to Graciá et al. [87], those samples (and the sampling site they represent) that have the lowest average *F*_ST_ and the highest allelic richness (*A*_*r*_) was regarded as those representing the likely starting point from which the species began colonizing, and samples (and the sampling site they represent) with the lowest average *F*_ST_ value was considered as those representing probable newly established derived populations occupying the new territory, or arrival sites. Range expansion is expected to cause a decrease in intrademe heterozygosity with an increase in *F*_ST_ as a function of the distance from the inferred starting point of colonization [88–91]. We regressed the *H*_*e*_, *A*_*r*_ and allele frequencies of each sampling site against the distance to the inferred starting or arrival sites within each cluster as inferred as described above from microsatellite data (Fig. 1b). Due to departures from IBD for the southeastern, northeastern and western clusters (Figs. 1b and 6), we conducted these analyses using the corresponding *F*_ST_ values instead of the geographic distance, as in such a case *F*_ST_ might more accurately reflect the dispersal distance between sites. Statistical analyses were performed using SPSS v.20.

## Results

### Genetic diversity of Brandt’s vole populations

No significant linkage disequilibrium was detected amongst any of the 12 pairwise microsatellite loci combinations. The genotyping results are listed in Additional file 1: Table S1, and characteristics of the microsatellite markers are summarized in Additional file 1: Table S2. The MICRO-CHECKER results suggested no notable scoring errors (the error rate of most loci was less than 0.05) due to stuttering or allele dropout. The number of alleles for each population across loci ranged from 3.83 (HW) to 8.17 (BY), with an average of 6.33. The mean observed heterozygosity (*H*_*o*_) and expected heterozygosity (*H*_*e*_) were 0.68 (0.54 for BL to 0.75 for AL) and 0.66 (0.55 for SH to 0.75 for AL), respectively. The mean allelic richness (*A*_*r*_) was 4.66 (3.39 for SH to 5.61 for AL) (Additional file 1: Table S3). Significant deviations from the Hardy-Weinberg expectation were found for 11 loci in some populations (*P* < 0.05) (Additional file 1: Table S4).

The analysis of D-loop sequences revealed 30 haplotypes based on 29 segregating sites (including insertions and deletions) observed in the 746 individuals sequenced. Of the 30 haplotypes, 14 were singletons, six were shared among individuals within geographical locations, and 10 were shared among different geographical locations (Additional file 1: Table S5). The global haplotype diversity (*Hd*) was 0.73, with a range from 0 for MD and TS to 0.83 for EH (Additional file 1: Table S6).

The Bayes tree and MJ network tree of D-loop haplotypes defined two haplogroups: hap_group1 (Hg1) and hap_group2 (Hg2) (Figs. 2 and 3a). Hg1 consisted of 18 haplotypes that are mainly found in the eastern distribution, and Hg2 consisted of 12 haplotypes that occurred mainly in the western distribution (Fig. 3b; Table 1). The predominant haplotypes (Hg1-H3 and Hg2-H5) were detected 288 and 247 times, respectively (Additional file 1: Table S5).

**Table 1:** Polymorphism and demographic statistics inferred from D-loop data for haplogroups in Brandt’s vole

Haplogroup	*n*	*N*	*Hd*	*π*	Tajima’s D	Fu’s	SSD	Raggedness
Hg1	418	18	0.5541	0.0385	−1.7622**	−18.5179**	0.007^ns^	0.1134**
Hg2	328	12	0.3171	0.0318	−1.9326**	−6.7297**	0.0003^ns^	0.2459^ns^
Overall	746	30	0.728	0.0021	−1.5135*	−18.5425**	0.013**	0.0531^ns^

*n* Sample size, *N* number of haplotypes, *Hd* haplotype diversity, *π* nucleotide diversity, *Tajima’s D* Tajima’s D value, *Fu’s* Fu and Li’s D value, *SSD* goodness-of-fit to a simulated population expansion, and *Raggedness* Harpending’s Raggedness index estimated under demographic expansion model. (ns: *P* > 0.05, *: *P* < 0.05, **: *P* < 0.01). (Hg1: H2, H3, H4, H6, H7, H9, H15, H16, H17, H18, H19, H21, H24, H25, H26, H27, H28, H29; Hg2: H1, H5, H8, H10, H11, H12, H13, H14, H20, H22, H23, H30) (Figs. 2 and 3a & b)

### Genetic structure of Brandt’s vole populations

The partitioning of the entire data set using the Bayesian method implemented in the STRUCTURE revealed the presence of two or three genetic clusters, with a maximum log likelihood of posterior probability [lnP(X/*K*) = −33,067] and maximum (Δ*K* = 226.2) at *K* = 2 (Additional file 2: Fig. S1). The sample sites at AL, BE, HH, BG, SH, TS and DR belong to a western cluster, in western Mongolia. The other locations grouped as eastern cluster were predominantly found in Inner Mongolia, China (Fig. 1b & c). With the exception that DR was partitioned into the eastern, the phylogenetic relationships were broadly consistent with NJ tree constructed from the microsatellite pairwise genetic distance (D_a_) matrix (Fig. 1a). At K = 3 (Additional file 2: Fig. S1), BT, HL, TM, DR and BO emerged as a distinct cluster in the northeastern region, and the other locations were divided into two clusters that geographically corresponded to the western (including MM, CG, HW, AL, BE, HH, BG, SH and TS) and southeastern distributions (including BL, AQ, QL, BX, MD, BY, EH, WL and TG) (Fig. 1b & d).

For all populations, a 9.02% of microsatellite variation was distributed among site populations (AMOVA, *P* < 0.0001). For the two clusters (partitioning AL, BE, HH, TS, BG and SH in the western distribution and the other populations in the eastern), 3.3% (*P* < 0.0001) of the total variation accounted for the among clusters (Fig. 1b; Table 2), whereas a smaller proportion (2.93%, *P* < 0.0001) of total variation emerged between clusters (F_CT_ = 0.33, *P* < 0.0001) if DR was assigned to the western cluster (Table 2). Based on the three genetic clusters identified by the analysis using STRUCTURE (Fig. 1b & d) as well as the results obtained during AMOVA for the two clusters (Table 2), we divided the 23 populations into 3 clusters (southeastern, northeastern and western distributions) (Fig. 1b). In this scenario, a 2.6% (*P* < 0.0001) of the total variation was assigned to the among clusters (Table 2). For the microsatellite data we considered a structure consisting of two and three clusters for modeling gene flow and verification of the genetic surfing theory in Brandt’s vole.

**Table 2:** AMOVA analysis of microsatellite and D-loop data in Brandt’s vole populations

	Microsatellite	*P*	D-loop	*P*
	Percentage of variation		Percentage of variation	
	23 populations		23 populations	
Among populations	9.02	0.000***	52.46	0.000***
Within populations	90.98		47.54	
	2 clusters		2 clusters	
Among clusters	3.3	0.000***	59.7	0.000***
Among populations within clusters	7.33	0.000***	7.24	0.000***
Within populations	89.38	0.000***	33.06	0.000***
	3 clusters		3 clusters	
Among clusters	2.6	0.000***	50.86	0.000***
Among populations within clusters	7.13	0.000***	8.90	0.000***
Within populations	90.27	0.000***	40.25	0.000***
	2 clusters (DR in the western)		2 haplogroups	
Among clusters/groups	2.93	0.000***	75.99	0.000***
Among populations within clusters /groups	7.46	0.000***	---	---
Within populations	89.61	0.000***	24.01	

For clusters (using microsatellite or D-loop) and haplogroup definitions see Figs. 1b, 2, 3c and Table 1, respectively. (ns: *P* > 0.05, *: *P* < 0.05, **: *P* < 0.01, ***P < 0.001)

The Bayes tree (Fig. 2) and MJ network (Fig. 3a) constructed from the D-loop data revealed a phylogeographic pattern where related haplogroups generally were distributed following our two-cluster model for D-loop sequences (Fig. 3b). Haplogroup 1(Hg1) consisted of 18 haplotypes associated primarily with the eastern region, whereas Hg2 associated mainly with the western region (Fig. 3b; Additional file 1: Table S5). Five haplotypes (H1, H20, H22, H23 and H30) in Hg2 were deviated from the pattern and mainly occurred in the eastern. However, except for H1 that was frequent (found in 27 samples) all others are singletons. All clades of *L. brandtii* in the Bayes tree give weight to a monophyletic species. Estimates of posterior probability in the Bayes tree (Fig. 2) support the monophyly of *L. brandtii*, consisting of haplogroups 1 and 2.

AMOVAs using D-loop data revealed that 52.46% (*P* < 0.0001) of the total variation was distributed among populations for all samples; 75.99% (*P* < 0.0001) of the total variation was distributed among the two haplogroups; and 59.7% (*P* < 0.0001) of the total variation was distributed between the eastern and western clusters, resulting in maximization of genetic differences (Fig. 3c; Table 2). Under the three clusters scenario a 50.86% (*P* < 0.001) of the total variation was distributed among clusters (Table 2). Divergence time estimation showed that Hg1 and Hg2 split occurred at about 0.053Mya (Million years age) with a 95% highest posterior density (HPD) of 0.017–0.088. The basal differentiation of Hg1 clade occurred at 0.041 Mya and 0.035 Mya for Hg2 (Fig. 2).

### Reconstruction of ancestral area and migration route

Based on the estimates of divergence time, Hg1(eastern) appears to have diverged earlier than Hg2 (Fig. 2). The ancestral areas inferred using S-DIVA and DEC were in broad agreement. The basal node 65 from S-DIVA displayed two possible ancestral ranges, SE (southeastern) and NE+ SE (northeastern + southeastern), and the probability of the two ancestral ranges were 63.5% and 20.9%, respectively (Fig. 3d; Additional file 1: Table S8). DEC yielded the same two ancestral ranges with S-DIVA, and the probability of these ranges at node 65 were 0.76 and 0.12 for SE and NE + SE, respectively (Additional file 1: Table S8). These results provided support for the ancestors of Brandt’s vole occurring in the southeastern portion of its distribution (Fig. 3c).

**Fig. 4:**
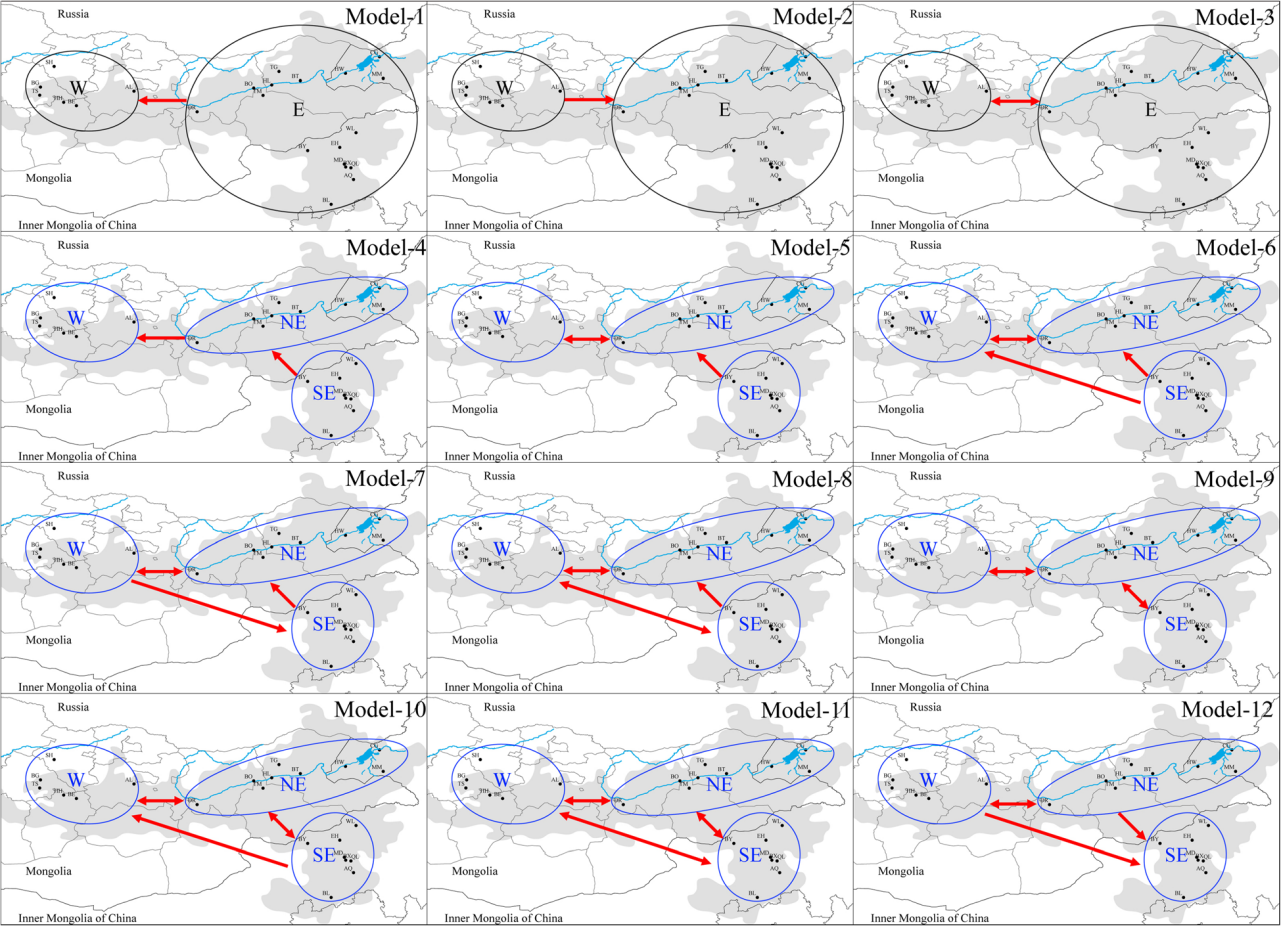
Full set of migration models (model 1–12) conceived and tested using microsatellite data simulate in the program MIGRATE-N. W, West; E, East; NE, Northeast; and SE, Southeast

We applied MIGRATE-N to the microsatellite data and found that Model 1 had the highest likelihood (Fig. 4; Table 3), thus consistent with the results for the D-loop data that Brandt’s vole originated from the eastern part of its current distribution (Fig. 3d). Under this scenario we evaluated nine migration models (Fig. 4). We found that Model 4 had the highest probability, implying that Brandt’s vole originated and spread from the southeast first in a northeastern direction and subsequently to the western ranges (Table 3). Notably, this Model 4 is somewhat unique in that it considered features of the landscape that may affect dispersal, i.e., the Gobi desert located at the border of Mongolia and Inner Mongolia of China [10, 92]. The other models were based on Model 4 but included additional direct migrations or backflows (Fig. 4).

**Table 3:** Marginal log-likelihoods and model probabilities for 15 migration models (Fig. 4) among two and three clusters using microsatellite data in Brandt’s vole

Regions	Number of samplings	Model	Bezier lmL	Harmonic lmL	Raw score	Mean lmL	Model probabilities
2 regions	5 × 10^7^	Model 1	−57,349.74	−1145.72	−316,290.09	−124,928.52	1
		Model 2	−63,735.82	−1364.26	−356,188.44	−140,429.51	0
		Model 3	−58,542.1	−1228.6	−335,142.1	−131,637.6	0
3 regions	5 × 10^7^	Model 4	−49,419.84	−1265.02	−262,401.1	−104,361.99	1
		Model 5	−50,478.96	−1223.96	−269,783.24	−107,162.05	0
		Model 6	−54,431.6	−993.38	−294,577.04	−116,667.34	0
		Model 7	−104,545.89	−1052.16	−607,229.48	−237,609.18	0
		Model 8	−118,766.8	−718.94	−696,727.88	−272,071.21	0
		Model 9	−172,482.06	−831.40	−1,032,522.62	−401,945.36	0
		Model 10	−108,659.74	−619.15	−634,189.74	−247,822.88	0
		Model 11	−1,683,126.9	−512.63	−276,531.64	−653,390.38	0
		Model 12	−94,635.4	−878.16	−545,702.85	−213,738.8	0

Model probabilities were calculated by Bezier lmL; lmL, log marginal likelihood

### Range expansion analysis of Brandt’s vole

**Fig. 5:**
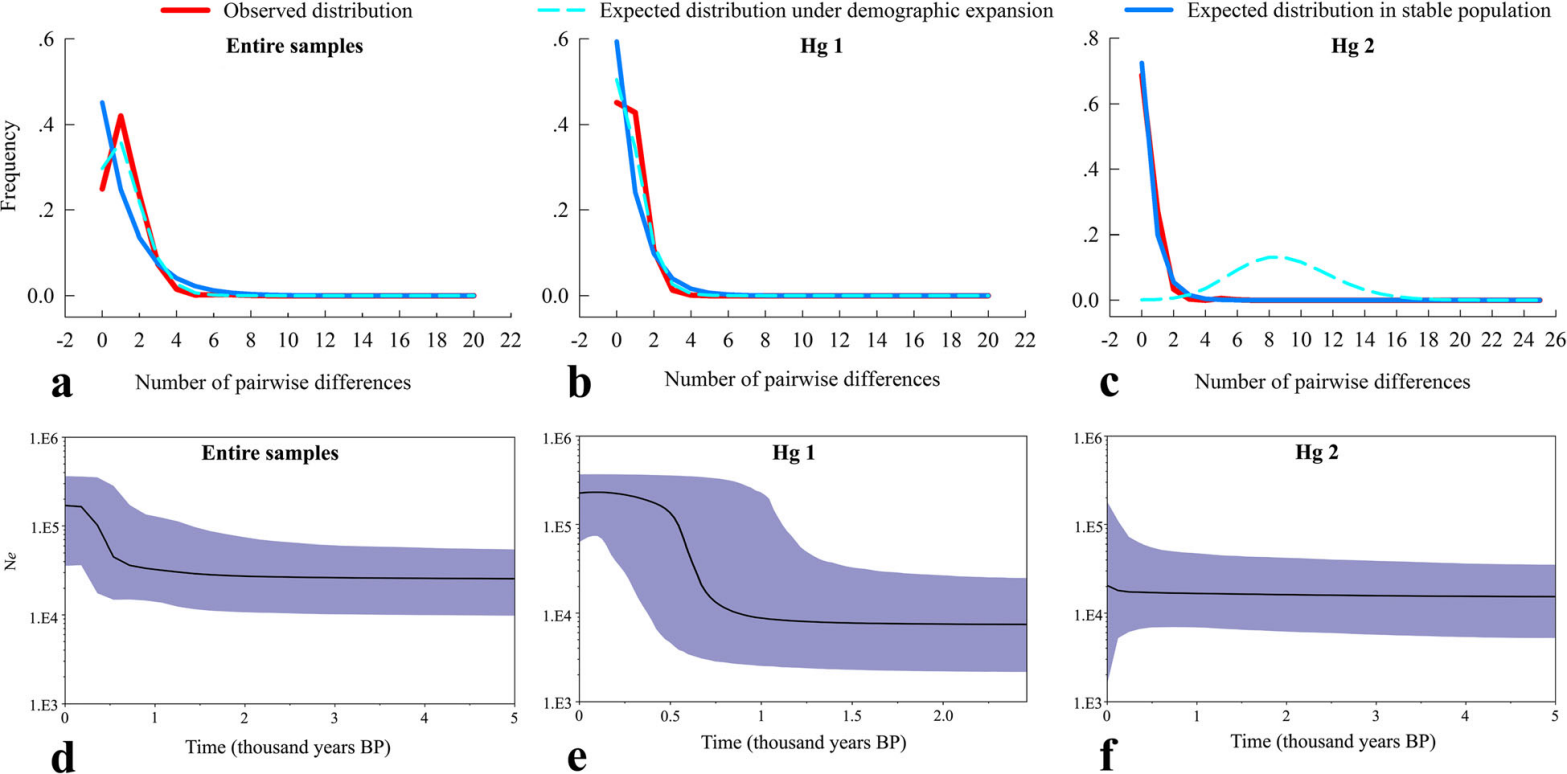
Mismatch distributions and Bayesian Skyline plots generated from D-loop data for Brandt’s voles. Mismatch distributions for the entire population (**a**) Hg1 (**b**) and Hg2 (**c**) (Table 1; Fig. 2). Effective population size fluctuations revealed by Bayesian Skyline plots for the entire population (**d**) Hg1 (**e**) and Hg2 (**f**). Middle line is the median estimate; blue shadow represents the 95% highest posterior density

We applied neutrality tests, mismatch distributions, and Bayes Skyline Plot (BSP) to the entire mtDNA data, and Hg1 and Hg2 (see above). The MJ network (Fig. 3a) already revealed a star-like pattern, with two centrally placed geographically widespread haplotypes H3 and H5 found in 19 and 13 sites throughout the eastern and western, respectively (Additional file 1: Table S5). For the entire population, Tajima’s D (−1.5135, *P* < 0.05) and Fu and Li’s F (*F* = −18.5425, *P* < 0.01) were both significantly negative (Additional file 1: Table S6), and the mismatch distributions were unimodal with low Harpending’s r (raggedness = 0.0531, *P* > 0.05) (Fig. 5a). When haplogroups were analyzed separately, the neutrality tests and mismatch distributions of Hg1 and Hg2 were consistent with rapid population size expansions (Fig. 5b & c; Table 1). The BSP also showed the signs of population expansion for the entire population as well as for Hg1 and Hg2, with an estimated time since population expansion for entire population of ~2000 years BP, and for Hg1 and Hg2 of ~1000 years BP and ~200 years BP, respectively (Fig. 5d–f). The lower diversity within Hg2 compared to Hg1 is consistent with such a more recent expansion (Table 1).

**Fig. 6:**
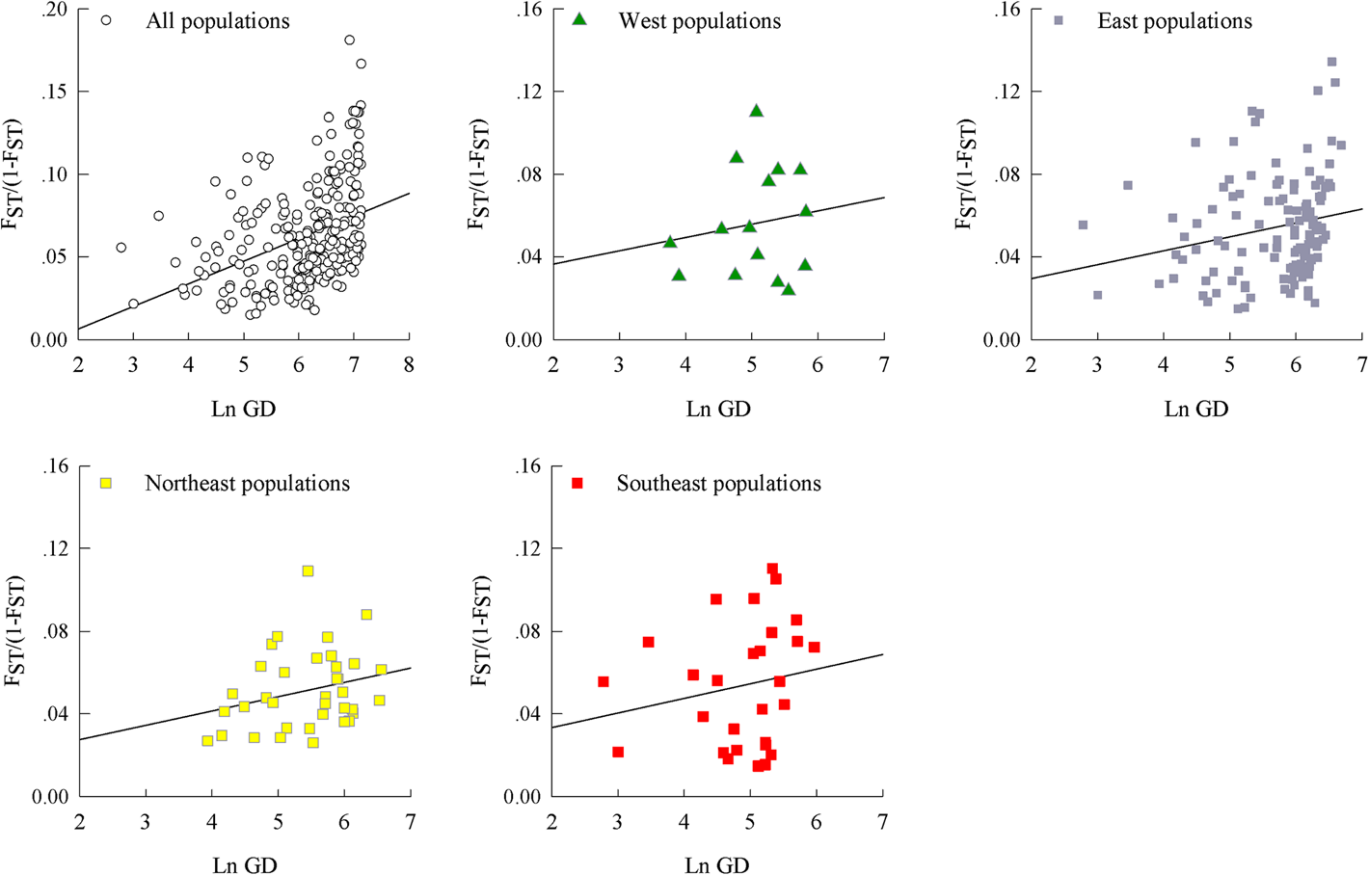
Tests of isolation by distance (IBD) analyses for each cluster (Fig. 1b) of Brandt’s vole using microsatellite data. The genetic distance measure *F*_ST_-(1- *F*_ST_) was plotted against the geographic distance measure (Ln GD, measured in km)

Average of *F*_*ST*_ values computed for the microsatellite data between sampling sites from the southeastern, northeastern and western range of the species varied, 0.042 (range 0.015–0.1), 0.044 (0.025–0.099), and 0.059 (0.023–0.099), respectively (Additional file 1: Table S7). *F*_*ST*_/ (1- *F*_*ST*_) and geographical distances (lnGD) were significantly correlated for the microsatellite dataset from entire samples (R^2^ = 0.149, *P* < 0.0001), which is consistent with IBD. Significant IBD was supported for the eastern samples if analyzed separately (R^2^ = 0.045, *P* = 0.021), but was not significant for samples from the western samples (R^2^ = 0.0645, *P* = 0.265). If we considered three, rather than two, regional genetically split populations none of the IBD analyses emerged as significant (southeast: R^2^ = 0.068, *P* = 0.126; northeast: R^2^ = 0.033, *P* = 0.354; west: R^2^ = 0.0645, *P* = 0.265) (Fig. 6).

Analysis of microsatellite data divided Brandt’s voles population into three clusters from southeastern, northeastern and western of its current distribution (Fig. 1b). Using this population structure and patterns of migration and population expansion we posited the hypothesis that the conditions were in place for gene surfing to have occurred in this species.

**Fig. 7:**
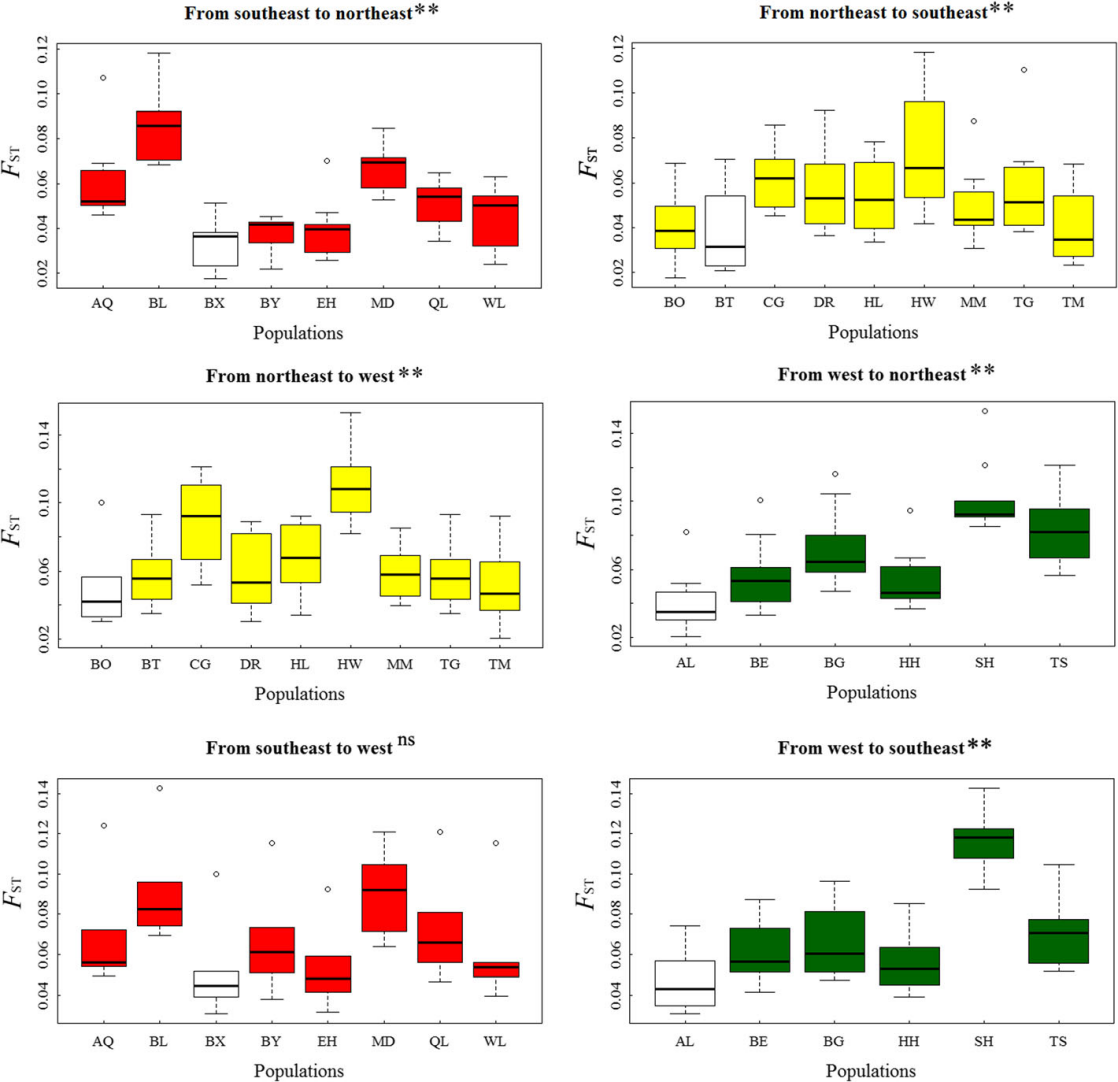
Pairwise *F*_ST_ values between each samples obtained from one site that is part of a cluster (c.f. Fig. 1b) and all samples from all sites from another cluster and estimated these values in a pairwise manner for the three clusters. The white column presents the population with the lowest average *F*_ST_ within the corresponding cluster, which is considered the likely starting or arrival site of Brandt’s vole. Population abbreviations as in Fig. 1b (ns: *P* > 0.05, *: *P* < 0.05, **: *P* < 0.01)

We obtained six sets of average *F*_ST_ values between one site samples in a cluster and the samples from each site in another cluster. ANOVA results revealed that BX population in the southeastern region had the significantly lowest value relative to all of the site samples in the northeastern region. Similar calculations revealed that the obviously lowest average *F*_ST_ was obtained for BT from the northeastern to the southeastern, BO from the northeastern to the western, AL from the western to both the northeastern and the southeastern, and BX had the lowest average *F*_ST_ from the southeastern to the western, although this value was not significant (Fig. 7). Based on the combination of the above-discribed results corresponding to the lowest average *F*_ST_ (Fig. 7) and genetic characteristics (Additional file 1: Table S3), we hypothesized that BX and BT were the starting and arrival sites of the population spreading from the southeasterm to the northeastern and that BO and AL were the starting and arrival sites of the population spreading from the northeastern to the western, even though BO did not present the highest genetic diversity.

**Fig. 8:**
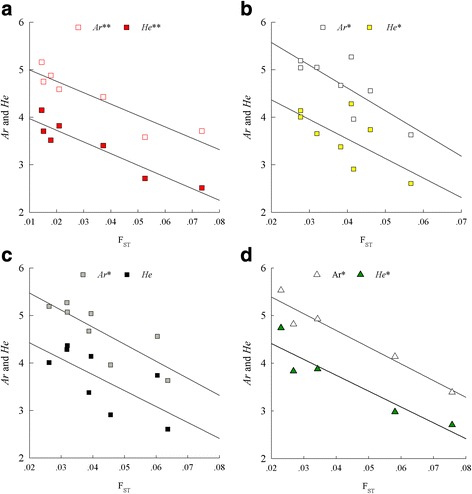
Regression of the allelic richness (*A*_*r*_) and expected heterozygosity (*H*_*e*_) of samples obtained from one site in a cluster (for clusters c.f. Fig. 1b) against *F*_ST_ values to the corresponding arrival or starting site (Fig. 7). Regressions are shown in **a** for each site samples in the southeastern cluster with regard to BX. **b** for each site samples in the northeastern cluster with BT. **c** for each site samples in the northeastern cluster with BO. **d** for each site samples in the western cluster with AL. (ns: * *P* < 0.05, ** *P* < 0.01)

In the three regions, the allelic richness (*A*_*r*_) and expected heterozygosity (*H*_*e*_) significantly decreased with increasing *F*_ST_ values to the probable start or arrival site with the expection of the starting site of BO in the northeastern (Fig. 8). Nine of all allele frequencies for samples from each site in the western significantly varied in relation to their *F*_ST_ to the arrival site AL (six decreased and three increased). Seven and five allele frequencies showed significant variations with increasing *F*_ST_ values to the probable arrival site BT and the starting site BO in the northeastern, respectively. In the southeastern, eight allele frequencies were significantly correlated with the *F*_ST_ to BX (Table 4). More clinal patterns of allele frequencies were observed in the colonization from the northeastern to the western but not in the expansion from the southeastern to the northeastern, which possibly resulted from the bridge status of the northeastern. Surprisingly, some alleles that were found to be rare in the sampling sites in southeastern and northeastern ranges showed high frequencies in some sites within the northeastern and western, respectively (Additional file 3: Table S9).

**Table 4:** Allele frequencies showing spatial clines in southeast, northeast and west clusters

Locus	allele	Southeastern (BX)		Northeastern (BT)		Northeastern (BO)		Western (AL)	
		frequency	spatial correlation (R^2^)	frequency	spatial correlation (R^2^)	frequency	spatial correlation (R^2^)	frequency	spatial correlation (R^2^)
DQ886928	183	0.311	n.s.	0.175	n.s.	0.175	n.s.	0.303	−0.738*
	185	0.140	n.s.	0.111	0.725*	0.111	n.s.	0.118	n.s.
FJ538254	258	0.457	0.68*	0.344	n.s.	0.344	n.s.	0.388	n.s.
	268	0.067	−0.828**	0.105	n.s.	0.105	n.s.	0.055	−0.889*
DQ886926	141	0.397	n.s.	0.289	n.s.	0.289	0.589*	0.462	−0.893*
DQ886929	177	0.537	n.s.	0.644	n.s.	0.644	n.s.	0.526	0.821*
DQ886927	152	0.309	n.s.	0.416	−0.506*	0.416	n.s.	0.291	n.s.
	182	0.081	n.s.	0.084	0.698**	0.084	0.541*	0.062	n.s.
DQ886925	164	0.633	−0.811**	0.246	0.45*	0.246	n.s.	0.585	0.95**
	168	0.191	0.72*	0.099	n.s.	0.099	n.s.	0.161	n.s.
	172	0.061	n.s.	0.011	n.s.	0.011	n.s.	0.093	0.729**
FJ538255	248	0.016	0.742*	0.047	n.s.	0.047	n.s.	0.007	n.s.
DQ886933	162	0.014	n.s.	0.045	n.s.	0.045	n.s.	0.030	−0.894*
	170	0.107	0.572*	0.075	n.s.	0.075	n.s.	0.022	−0.72*
	172	0.307	0.85**	0.049	n.s.	0.049	n.s.	0.186	n.s.
	174	0.084	n.s.	0.084	−0.48*	0.084	n.s.	0.145	n.s.
	186	0.048	n.s.	0.063	n.s.	0.063	−0.518*	0.089	n.s.
DQ886931	219	0.145	n.s.	0.124	n.s.	0.124	0.516*	0.220	−0.764*
FJ538252	296	0.439	n.s.	0.431	0.716**	0.431	n.s.	0.258	n.s.
	300	0.210	0.637*	0.233	−0.676*	0.233	−0.663*	0.248	n.s.

Southeast allele frequencies were correlated with *F*_ST_ to the putative starting site (BX), while northeast allele frequencies were correlated with *F*_ST_ to the probable arrival site (BT); northeast and west allele frequencies were correlated with *F*_ST_ values to the starting site (BO) and the arrival site (AL), respectively (ns: *P* > 0.05, *: *P* < 0.05, **: *P* < 0.01) ﻿

## Discussion

Brandt’s vole is a species of *Lasiopodomys* that was recently separated from the genus *Microtus* [93]. The species has some peculiar biological and ecological characteristics, i.e., seasonal and multi-annual population fluctuations [17, 18] and the discrete and homogeneous habitat environment. Our work described here is an attempt to re-construct the geographic origin of the species in current distribution and subsequent events that led to the present-day distribution of the species. In addition, during this study the phenomenon of genetic surfing emerged as a possible explanation for the peculiar patterns of genetic differentiation, and the success of genetic lineages.

### Ancestral area of Brandt’s vole

Phylogeographic and STRUCTURE analysis of microsatellite data and D-loop sequences revealed that Brandt’s vole currently is comprised out of two to three genetically differentiated populations (Figs. 2 and 3b). To refer to the spatial structuring of the species we refer to these as eastern and western clusters (two symbols in Figs. 1b and 3c), or if we consider are more refined structuring we refer to these as southeastern, northeastern and western clusters (two symbols in Figs. 1b and 3c). We used the three clusters in the analyses of origination and colonization route, the southeastern cluster presented the maximum probability ancestry (*P* = 63.5% for S-DIVA and *P* = 0.76 for DEC) of the most primitive ancestral haplotype inferred by RASP and Lagrange (Fig. 3d; Additional file 3: Table S9). These results strongly supported the southeastern region (specifically Xilinhaote District, Inner Mongolia of China) as being the ancestral location of Brandt’s vole prior to its expansion (Fig. 3c). This coincides with the pleistocene fossil evidence observed in northern China [10].

### Range expansion of Brandt’s vole

Neutral tests (Table 1), mismatch analysis (Fig. 5) and MJ network (Fig. 3b; Additional file 1: Table S5) of D-loop sequences indicating a past rapid expansion of the population of Brandt’s vole from a few founders [83, 89, 94, 95]. The diversity values (Table 1), mismatch distributions (Fig. 5b & c) and BSP (Fig. 5e & f) revealed that Hg2 expanded later than Hg1, with the temporal scale and spatial scope essentially agreeing with the optimal colonization route, which was from the ancestral area (the southeastern) to the western through the northeastern (Fig. 4). These findings supported a scenario where a small number of founders disperse and initiate colonization followed by a rapid population expansion [80]. Pleistocene fossils of the species further indicate that its previous distribution was wider than the current one [10]. Thus, the molecular data and the fossil evidence remain to be reconciled, the fact that the past distribution was wider than the present distribution implies that the populations spread from their initial location.

Range expansion can be viewed as a series of successive founder events [88] that result in a decrease in intrademe heterozygosity with increasing distance from the ancestral population to other demes within a region [88–91]. The IBD for all Brandt’s vole populations showed that the population expansion generally satisfied the one-dimensional stepping-stone model (R^2^ = 0.149, *P* < 0.0001) [85]. However, none of the regressions for the southeastern, northeastern and western were significant, which suggests the possible existence of geographic barriers or a greatly heterogeneous environment. Therefore, we used pairwise *F*_ST_ values instead of the geographical distance to test the range expansion of Brandt’s vole. The results that the *H*_*e*_ values significantly decrease with increasing *F*_ST_ values to the probable arrival (BT and AL) or starting (BX and BO) sites for the species (Fig. 8) supported the hypothesis of range expansion.

The fossil evidence indicates that *Lasiopodomys* was present in the late Early Pleistocene [10]. We inferred the expansion time of *L.brandtii* happed earlier than 0.041 Mya (about middle-late Pleistocene), which is for the species’ diversion into Hg1 clade (Fig. 2). In this period, the climate changed towards cooler and more arid, causing land degeneration and desertification [28, 29], big area of meadow-steppes degenerated gradually into the dry steppes and semi-deserts in Mongolia [3, 10, 96], where were favourable habitats for Brandt’s voles. That might have promoted the dispersal and increase in the species populations over time [17, 24]. Towards the Holocene, the climate gradually became mild, resulting expanding forest in the west and central Mongolia [25] and the Transbaikal plains, Russia [10]. As a result, the suitable habitats of Brandt’s vole significantly decreased. The changes would destroy some preferred habitats of Brandt’s vole and cause fragmentation and reduction in their distribution [97], which possibly contributed to the differences between the populations from the original and colonized regions.

### Genetic surfing in Brandt’s vole population

Prior to this study, the genetic dynamics in range expansions have mostly been based on modeling, simulations and microcosm experiments and have not been corroborated using field data. In accordance with the colonization route of Brandt’s vole from the southeastern to the western through the northeastern, the neutral genetic pattern of the population matches the predictions of the genetic surfing theory:

1. A steady reduction in genetic diversity with increasing *F*_ST_ to the starting site centroid (BX and BO) or the probable arrival site centroid (BT and AL) within each of the three regions (Fig. 8) [87].
2. The alleles present on the wave front of an expansion could increase in frequency and reach very high proportions and even fixations far away from their original areas [39, 98, 99], and this change is particularly observed in the frequencies of rare alleles [100]. In the surveyed Brandt’s vole populations, some rare alleles in both the southeastern and the northeastern have become common in the corresponding expansion ranges, namely the northeastern and western (Additional file 3: Table S9). Additionally, more clinal patterns of allele frequencies involving different loci are found in the colonized areas compared with the original areas (Table 4), although this difference is not significant.
3. The spatial differentiation is stronger in the more recently established range than in the original one [101], and this finding is also supported by the mean pairwise *F*_ST_ values within each region calculated from microsatellite data, which were found to equal 0.042, 0.044 and 0.059 for the western, southeastern and northeastern, respectively (Additional file 1: Table S7).

Genetic drift acting on the advancing front could cause the extant spatial genetic patterns of Brandt’s vole during its expansion within the distribution. Some individuals, particularly those in an outbreak population, possibly disperse outward from the original habitat due to intraspecific competition. These voles were likely the pioneers in the new habitats. Such dispersal can lead to large gene frequency changes and can determine the genetic diversity of colonies propagated by aggressors [101], as was typically observed in the populations from BX in the southeastern to BT in the northeastern, and from BO in the northeastern to AL in the western (Fig. 1b). Consequently, this distribution would lead to increased global genetic differentiation and strengthen the presence of migration among populations [91, 99, 101, 102]. These patterns are also supported by the results of genetic structure analysis (Fig. 1; Table 2) and the most likely migration models (Fig. 5; Table 3) inferred from microsatellite data.

The spatial genetic patterns of Brandt’s vole populations are unlikely to have been produced by two important alternative scenarios: adaptive events generated by selective sweeps [27, 103] and introgression [104, 105]. For example, clines in allele frequency are often attributed to geographic variations in selection intensity [91]. Although Brandt’s vole distribution was composed of many patchy homogeneous habitats [20], the geographic variation in selection intensity was low. Genetic patterns such as gradients of introgression [104, 105] or bidirectional introgression close to the introduction area of an invasive species are often interpreted as footprints of selection [27]. Introgression generally occurs in inter-divergent geographical populations, and the divergence level of the two/three clusters in Brandt’s vole population did not support the taxonomy of subspecies (Fig. 1a).

A combination of demographic properties including population size, growth rate and migration rate, determines the success of mutations in populations under range expansion [39, 106, 107]. Our results illustrate the existence of the surfing phenomenon during Brandt’s vole range expansion, but the demographic properties of the successful colonized populations, such as the populations around BX and BO sites, require further investigation. Otherwise, the heterogeneous landscape might play an important role in determining the fate of mutations, such as their locations [108, 109]. It is undoubtedly more complex than corroborating the effects of these processes in natural populations.

### Hypothesis of colonization pattern in Brandt’s vole

In a patch structure of an environment, the habitat types selected by individuals should be a combined result of the intrinsic “quality” of the habitat type and the population density (intensity of competition) in the habitat [110, 111]. Population outbreaks of Brandt’s voles occur irregularly, with an interval of five to seven years [17, 18]. Based on repetitions of this pattern of spatial change throughout history, an outbreak population with opportunistic demographic properties, i.e., suitable population size, growth rate and migration rate, inhabiting the surroundings of the BX site located in the current southeastern distribution was expanding spatially. Some “excess” individuals arrived and successfully settled in a new habitat in the area surrounding the BT site, which was never previously occupied by the species and that is equal in quality to the original habitat. These settlers acted as a seed for the population colonizing the new territories located in the current northeastern distribution. Similar processes occurred during the colonization from BO site in the northeastern to AL site in the western (Fig. 1c) and perhaps further westward until Brandt’s vole population covered the whole distribution area. Such expansion processes can lead to the spatial patterns of genetic structure coinciding with the traits of the genetic surfing phenomenon [39, 101, 112]. Our results from genetic data match the predictions made based on the genetic surfing theory.

## Conclusions

We hypothesized that Brandt’s voles originated from the extant southeastern distribution. Under the integrative effect of various factors, such as temperature, rainfall, vegetation and landscape traits, this species colonized from the original area surrounding BX in Xilinguole region, propagating by wave within the region to reach the probable arrival site BT in the northeastern and expanding thereafter within the area; the subsequent expansion of Brandt’s voles population to the western at the arrival site AL from the starting site BO in the northeastern followed the same surfing pattern. Landscape heterogeneity, a low density of individuals in the front of the wave and a low dispersal capacity promote the strong genetic structuration of the three clusters. Brandt’s vole thus formed the current patterns, which are characterized by isolated, patchy, unstable habitats with some genetic characteristics.

## Additional files

Additional file 1: Table S1. Sampling information for Brandt’s vole. **Table S2.** Summary statistics for twelve microsatellite loci over all Brandt’s vole samples. **Table S3.** Genetic diversity measures calculated over 12 microsatellite loci and across all geographic locations. **Table S4.** Summary of Chi-Square Tests for Hardy-Weinberg Equilibrium for each geographic location sampled. **Table S5.** Geographic occurrences and frequencies of D-loop haplotypes H1–30. **Table S6.** Summary statistics on polymorphism and demographics inferred from analyses of D-loop sequences. **Table S7.** Pairwise *F*_*ST*_ values calculated based on microsatellite data between 23 geographic locations sampled. **Table S8.** Clades support and ancestral areas reconstruction as obtained with S-DIVA and Lagrange. (DOCX 115 kb)

Additional file 2: Figure S1. Brandt’s vole population structure based on 12 microsatellites loci as implemented by STRUCTURE. A) Values of lnP(D) from 20 independent runs for K = 1–23. B) Values of ΔK from 20 independent runs plotted for K = 1–23 and calculated by the Evanno method. (PDF 3121 kb)

Additional file 3: Table S9. A) Allele frenquency of each locus for Brandt’s vole populations in southeastern and northeastern distribution; B) Allele frenquency of each locus for Brandt’s vole populations in northeastern and western distribution. (XLSX 62 kb)
