# Supplementary material for: The colonization and divergence patterns of Brandt’s vole (Lasiopodomys brandtii) populations reveal evidence of genetic surfing
Source: BMC Evol Biol. 2017 Jun 21;17:145. doi: 10.1186/s12862-017-0995-y (PMC5480173; doi:10.1186/s12862-017-0995-y)
Supplement: Supplementary file 1 — Sampling information for Brandt’s vole. Table S2. Summary statistics for twelve microsatellite loci over all Brandt’s vole samples. Table S3. Genetic diversity measures calculated over 12 microsatellite loci and across all geographic locations. Table S4. Summary of Chi-Square Tests for Hardy-Weinberg Equilibrium for each geographic location sampled. Table S5. Geographic occurrences and frequencies of D-loop haplotypes H1–30. Table S6. Summary statistics on polymorphism and demographics inferred from analyses of D-loop sequences. Table S7. Pairwise F ST values calculated based on microsatellite data between 23 geographic locations sampled. Table S8. Clades support and ancestral areas reconstruction as obtained with S-DIVA and Lagrange. (DOCX 115 kb) [file 12862_2017_995_MOESM1_ESM.docx]

**Supporting Material for:**

**The** **colonization and divergence patterns of Brandt’s vole (*Lasiopodomys brandtii*) populations reveal evidence of genetic surfing**

Ke Li^1^, Michael H. Kohn^2^, SongMei Zhang^1^, XinRong Wan^3^, DaZhao Shi^1^, Deng Wang^1^*

**Supplementary Tables**

**Table S1** Sampling information for Brandt’s vole

| Country | Locality | Site code | Coordinate | Sample size | | |
| --- | --- | --- | --- | --- | --- | --- |
|  |  |  |  | N | nSSR | mtDNA |
| Inner Mongolia,  China | Baoligentaohai | BL | 42°25.61' N;115°16.33' E | 27 | 27 | 25 |
|  | Aqiwula | AQ | 43°25.64´N;116°51.31´E | 30 | 29 | 27 |
|  | Qilianhaote | QL | 43°58.96´N;116°43.81´E | 30 | 30 | 26 |
|  | Baiyinxile | BX | 44°01.34´N;116°29.07´E | 31 | 31 | 31 |
|  | Maodeng | MD | 44°09.51´N;116°24.95´E | 30 | 30 | 30 |
|  | Baiyantuga | BY | 44°52.572´N;114°41.716´E | 40 | 38 | 34 |
|  | Ehebaolige | EH | 44°59.453´N;116°10.847´E | 35 | 33 | 34 |
|  | Wuliyasitai | WL | 45°39.37´N; 116°58.52´E | 41 | 41 | 39 |
|  | Hangwula | HW | 48°28.21´N; 116°28.48´E | 10 | 10 | 8 |
|  | Modamuji | MM | 48°13.852´ N; 118°15.642´E | 10 | 8 | 7 |
|  | Cuogang | CG | 49°18.153´N; 118°03.777´E | 57 | 29 | 54 |
| Mongolia | Bayanovoo | BO | 47°46.746´N; 112°07.220´E | 29 | 29 | 26 |
|  | Tsagaanovoo | TG | 48°33.288´N; 113°19.053´E | 36 | 36 | 29 |
|  | Bayantumen | BT | 48°07.568´N; 114°19.865´E | 45 | 45 | 29 |
|  | Hulunbuir | HL | 47°55.464´N;112°58.548´E | 43 | 43 | 32 |
|  | Tumentsogt | TM | 47°25.722´N; 112°33.621´E | 53 | 53 | 40 |
|  | Darkhan | DR | 46°39.018´N; 109°26.161´E | 32 | 32 | 30 |
|  | Altan-bulag | AL | 47°37.898´N; 106°26.374´E | 56 | 56 | 52 |
|  | Burd | BE | 46°56.294´N; 103°42.792 ´E | 41 | 41 | 35 |
|  | Kharhorin | HH | 47°05.971´N; 103°06.431´E | 26 | 25 | 21 |
|  | Tovshruuleh | TS | 47°26.330´N; 101°58.040´E | 55 | 55 | 51 |
|  | Battsengel | BG | 47°49.572´N; 102°00.508´E | 63 | 63 | 56 |
|  | Saihan | SH | 48°47.594´N; 102°39.279´E | 31 | 30 | 30 |
|  | Total | 23 | --------- | 851 | 814 | 746 |

**Table S2** Summary statistics for twelve microsatellite loci over all Brandt’s vole samples

| Locus | N | *N*_a_ | *N*_e_ | *H*_e_ | *H*_o_ | PIC | F(null) |
| --- | --- | --- | --- | --- | --- | --- | --- |
| DQ886925 | 812 | 12 | 2.514 | 0.560 | 0.546 | 0.650 | 0.0487 |
| DQ886926 | 813 | 11 | 3.595 | 0.692 | 0.713 | 0.761 | 0.0389 |
| DQ886927 | 813 | 25 | 4.932 | 0.764 | 0.788 | 0.813 | 0.0302 |
| DQ886928 | 814 | 13 | 4.087 | 0.736 | 0.772 | 0.797 | 0.0309 |
| DQ886929 | 814 | 10 | 2.417 | 0.560 | 0.579 | 0.543 | 0.0267 |
| DQ886931 | 814 | 6 | 2.175 | 0.500 | 0.473 | 0.519 | 0.0416 |
| DQ886932 | 812 | 12 | 2.067 | 0.478 | 0.495 | 0.479 | 0.0393 |
| DQ886933 | 812 | 25 | 6.024 | 0.806 | 0.827 | 0.908 | 0.0297 |
| FJ538247 | 812 | 10 | 3.670 | 0.715 | 0.745 | 0.781 | 0.0401 |
| FJ538252 | 812 | 8 | 3.495 | 0.697 | 0.744 | 0.733 | 0.0234 |
| FJ538254 | 814 | 17 | 4.234 | 0.722 | 0.739 | 0.773 | 0.0337 |
| FJ538255 | 813 | 16 | 4.489 | 0.750 | 0.791 | 0.818 | 0.0203 |
| Mean | ------ | 13.75 | 3.642 | 0.665 | 0.684 | 0.715 | ------ |

N, the number of individuals; *N*_a_, total number of alleles; *N_e_*, number of effective alleles; *H*_o_, the observed heterozygosity; *H*_e_, the expected heterozygosity; PIC, polymorphism information content; F(null), the frequency of null alleles.

**Table S3** Genetic diversity measures calculated over 12 microsatellite loci and across all geographic locations.

| Populations | *Samples no* | *Na* | *Ne* | *Ar* | *PAr* | *Ho* | *He* | TPM (P) | *F_IS_* |
| --- | --- | --- | --- | --- | --- | --- | --- | --- | --- |
| BL | 27 | 5.167 | 2.514 | 3.71 | 0.09 | 0.542* | 0.577 | 0.8303 | 0.0682 |
| AQ | 29 | 6 | 3.406 | 4.43 | 0.08 | 0.669 | 0.647 | 0.78809 | -0.0196 |
| QL | 30 | 5.917 | 3.821 | 4.59 | 0.05 | 0.697* | 0.649 | 0.2347 | -0.057 |
| BX | 31 | 7.833 | 3.954 | 5.25 | 0.13 | 0.71 | 0.695 | 0.9933 | -0.0044 |
| MD | 30 | 4.25 | 2.712 | 3.58 | 0.19 | 0.686** | 0.605 | 0.4251 | -0.1166 |
| BY | 38 | 8.167 | 4.149 | 5.16 | 0.09 | 0.655* | 0.69 | 0.9614 | 0.0563 |
| EH | 33 | 7.167 | 3.516 | 4.88 | 0.05 | 0.673 | 0.671 | 0.9451 | 0.0111 |
| WL | 41 | 6.75 | 3.709 | 4.75 | 0.05 | 0.634 | 0.657 | 0.7881 | 0.0465 |
| HW | 10 | 3.833 | 2.606 | 3.63 | 0.13 | 0.733* | 0.593 | 0.9829 | -0.1874 |
| MM | 8 | 4.667 | 3.377 | 4.67 | 0.13 | 0.707 | 0.658 | 0.3667 | -0.0174 |
| CG | 29 | 5.75 | 3.739 | 4.56 | 0.05 | 0.724 | 0.697 | 0.968 | -0.022 |
| BO | 29 | 6.833 | 3.656 | 5.05 | 0.02 | 0.707 | 0.684 | 0.311 | -0.0155 |
| TG | 36 | 5.167 | 2.909 | 3.96 | 0.04 | 0.685 | 0.622 | 0.6614 | -0.088 |
| BT | 45 | 7.25 | 4.366 | 5.07 | 0.15 | 0.722** | 0.698 | 0.8303 | -0.0241 |
| HL | 43 | 6.5 | 4.139 | 5.04 | 0.04 | 0.752* | 0.723 | 0.1506 | -0.0283 |
| TM | 53 | 7.5 | 4.007 | 5.19 | 0.04 | 0.73 | 0.729 | 0.6613 | 0.0092 |
| DR | 32 | 6.917 | 4.285 | 5.27 | 0.24 | 0.724 | 0.736 | 0.3667 | 0.0322 |
| AL | 56 | 8.083 | 4.756 | 5.61 | 0.11 | 0.753* | 0.755 | 0.3667 | 0.0123 |
| BE | 41 | 6.667 | 3.832 | 4.82 | 0.02 | 0.713 | 0.694 | 0.6045 | -0.0156 |
| HH | 25 | 7.167 | 4.742 | 5.53 | 0.12 | 0.736 | 0.725 | 0.3667 | 0.001 |
| TS | 55 | 6.083 | 2.979 | 4.14 | 0.06 | 0.548** | 0.583 | 0.9539 | 0.0689* |
| BG | 63 | 7.667 | 3.879 | 4.93 | 0.09 | 0.689 | 0.654 | 0.9739 | -0.0458 |
| SH | 30 | 4.333 | 2.704 | 3.39 | 0.02 | 0.546 | 0.552 | 0.4848 | 0.0251 |
| Average | ---- | 6.333 | 3.642 | 4.66 | 0.087 | 0.684 | 0.665 | 0.6528 | -0.0135 |

*N_a_*, number of alleles; *N_e_*, number of effective alleles; *A_r_*, allelic richness; *PA_r_*, private allelic richness; *H_o_*, observed heterozygosity; *H_e_*, expected heterozygosity; TPM (P), the probabilities from one-tailed Wilcoxon signed-rank tests of population bottlenecks; and *F_IS_*, inbreeding cofficients; Significant departure from HWE for each population over loci at 0.05 and 0.01 levels are denoted by * and **, respectively.

**Table S4** Summary of Chi-Square Tests for Hardy-Weinberg Equilibrium for each geographic location sampled

| Sites | BL | AQ | QL | BX | MD | BY | EH | WL | HW | MM | CG | BO | TG | BT | HL | TM | DR | AL | BE | HH | TS | BG | SH | Total |
| --- | --- | --- | --- | --- | --- | --- | --- | --- | --- | --- | --- | --- | --- | --- | --- | --- | --- | --- | --- | --- | --- | --- | --- | --- |
| Loci/N | 27 | 29 | 30 | 31 | 30 | 38 | 33 | 41 | 10 | 8 | 29 | 29 | 36 | 45 | 43 | 53 | 32 | 56 | 41 | 25 | 55 | 63 | 30 | 814 |
| DQ886928 | ns | ns | ns | ns | ns | ns | ns | ns | ns | ns | ns | ns | ns | *** | ns | ns | ns | ns | ns | ns | ns | * | ns | *** |
| FJ538254 | *** | ns | ns | ns | ns | ns | ns | ns | ** | ns | *** | ns | *** | *** | ns | *** | ns | ns | ns | ns | *** | *** | ns | *** |
| FJ538247 | *** | ns | ns | ns | * | *** | ns | ns | ns | ns | *** | ns | * | ns | * | ns | ns | * | ns | ns | ns | ns | ns | *** |
| DQ886926 | ns | ns | ns | ns | ns | ns | ns | ns | ns | ns | ns | ns | ns | ns | ns | ns | ns | ns | ns | ns | ns | ** | ns | *** |
| DQ886929 | ns | * | ns | ns | ns | ns | ns | ns | ns | ns | ns | ns | ns | ns | * | ns | ns | ns | *** | ns | ns | ns | ns | ns |
| DQ886927 | * | *** | ns | *** | ns | ** | *** | ns | ns | ns | ns | ** | ns | ns | *** | ns | ns | *** | ns | * | ns | ns | ns | *** |
| DQ886932 | ns | ns | ns | *** | ns | ns | ns | ns | ns | ns | ns | ns | ns | ns | ns | ns | ns | *** | ns | *** | ns | ns | ns | *** |
| DQ886933 | ns | ns | ns | * | *** | *** | ns | ** | ns | ns | ns | ns | * | * | *** | ns | ns | ns | ns | * | ** | *** | ns | *** |
| DQ886931 | * | * | ns | *** | ns | *** | ** | *** | * | ns | * | ns | *** | *** | *** | *** | *** | ns | ns | ns | * | ns | ns | *** |
| FJ538252 | ** | ns | ns | ns | * | ns | ns | ns | ns | ns | ns | ns | * | ns | *** | ns | ns | ns | ns | ns | ** | ns | ns | *** |
| DQ886925 | ns | ns | ns | ns | ns | *** | ns | ns | ns | ns | ns | ns | ns | ns | ns | ns | * | *** | ns | ** | * | ns | ** | *** |
| FJ538255 | ns | ns | ns | *** | * | ns | ns | ns | ns | ns | * | ns | ns | ns | * | * | ns | * | ** | ns | * | ns | ** | *** |

ns: not significant, * P<0.05, ** P<0.01, *** P<0.001

**Table S5** Geographic occurrences and frequencies of D-loop haplotypes H1-30

| populations | N= | H1 | H2 | H3 | H4 | H5 | H6 | H7 | H8 | H9 | H10 | H11 | H12 | H13 | H14 | H15 | H16 | H17 | H18 | H19 | H20 | H21 | H22 | H23 | H24 | H25 | H26 | H27 | H28 | H29 | H30 |
| --- | --- | --- | --- | --- | --- | --- | --- | --- | --- | --- | --- | --- | --- | --- | --- | --- | --- | --- | --- | --- | --- | --- | --- | --- | --- | --- | --- | --- | --- | --- | --- |
| BL | 25 |  |  | 24 |  |  |  |  |  |  |  |  |  |  |  |  |  |  |  |  |  |  |  |  |  | 1 |  |  |  |  |  |
| AQ | 27 |  | 1 | 26 |  |  |  |  |  |  |  |  |  |  |  |  |  |  |  |  |  |  |  |  |  |  |  |  |  |  |  |
| QL | 26 | 3 | 1 | 22 |  |  |  |  |  |  |  |  |  |  |  |  |  |  |  |  |  |  |  |  |  |  |  |  |  |  |  |
| BX | 31 |  | 4 | 26 |  |  |  | 1 |  |  |  |  |  |  |  |  |  |  |  |  |  |  |  |  |  |  |  |  |  |  |  |
| MD | 30 |  |  | 30 |  |  |  |  |  |  |  |  |  |  |  |  |  |  |  |  |  |  |  |  |  |  |  |  |  |  |  |
| BY | 34 |  | 11 | 12 |  | 1 | 1 |  | 3 |  |  |  |  |  | 2 | 2 |  |  |  |  |  |  |  |  |  |  | 1 | 1 |  |  |  |
| EH | 34 |  | 3 | 13 |  | 4 | 2 |  | 3 |  |  | 1 |  |  | 2 | 2 |  |  |  |  |  |  |  |  |  |  |  |  | 2 | 1 | 1 |
| WL | 39 |  | 10 | 2 |  |  | 26 |  | 1 |  |  |  |  |  |  |  |  |  |  |  |  |  |  |  |  |  |  |  |  |  |  |
| HW | 8 |  |  | 6 |  |  |  |  |  | 2 |  |  |  |  |  |  |  |  |  |  |  |  |  |  |  |  |  |  |  |  |  |
| MM | 7 |  |  | 4 | 1 | 1 | 1 |  |  |  |  |  |  |  |  |  |  |  |  |  |  |  |  |  |  |  |  |  |  |  |  |
| CG | 54 |  |  | 52 |  |  | 1 |  | 1 |  |  |  |  |  |  |  |  |  |  |  |  |  |  |  |  |  |  |  |  |  |  |
| BT | 29 |  | 14 | 1 |  | 1 | 7 |  | 1 |  |  |  |  |  |  |  |  |  | 1 | 4 |  |  |  |  |  |  |  |  |  |  |  |
| TG | 29 |  |  | 21 |  |  | 2 |  |  |  |  |  |  |  |  | 2 | 3 | 1 |  |  |  |  |  |  |  |  |  |  |  |  |  |
| BO | 26 | 2 |  | 15 |  | 1 |  |  | 3 |  |  | 3 |  |  |  | 2 |  |  |  |  |  |  |  |  |  |  |  |  |  |  |  |
| TM | 40 | 18 | 1 | 11 |  |  | 8 |  |  |  |  |  |  |  |  |  |  |  |  |  |  |  | 1 | 1 |  |  |  |  |  |  |  |
| HL | 32 |  |  | 13 |  | 13 | 4 |  |  |  |  |  |  |  |  |  |  |  |  |  | 1 | 1 |  |  |  |  |  |  |  |  |  |
| DR | 30 |  |  |  |  | 23 | 4 |  | 1 |  |  |  |  |  | 1 |  |  |  |  |  |  |  |  |  | 1 |  |  |  |  |  |  |
| AL | 52 |  |  |  |  | 37 |  |  |  |  | 2 | 6 | 4 | 3 |  |  |  |  |  |  |  |  |  |  |  |  |  |  |  |  |  |
| BE | 36 |  |  | 7 |  | 27 |  |  | 1 |  |  |  |  |  |  |  |  |  |  |  |  |  |  |  |  |  |  |  |  |  |  |
| HH | 21 |  |  | 1 |  | 13 |  |  |  |  |  | 1 | 6 |  |  |  |  |  |  |  |  |  |  |  |  |  |  |  |  |  |  |
| TS | 51 |  |  |  |  | 51 |  |  |  |  |  |  |  |  |  |  |  |  |  |  |  |  |  |  |  |  |  |  |  |  |  |
| BG | 56 | 4 |  | 2 |  | 46 |  |  |  |  |  | 1 |  |  | 2 |  |  |  |  |  |  |  |  |  |  |  |  |  |  |  |  |
| SH | 31 |  |  |  |  | 29 |  |  |  |  |  |  | 2 |  |  |  |  |  |  |  |  |  |  |  |  |  |  |  |  |  |  |
| sum | 746 | 27 | 45 | 288 | 1 | 247 | 56 | 1 | 14 | 2 | 2 | 12 | 12 | 3 | 7 | 8 | 3 | 1 | 1 | 4 | 1 | 1 | 1 | 1 | 1 | 1 | 1 | 1 | 2 | 1 | 1 |

**Table S6** Summary statistics on polymorphism and demographics inferred from analyses of D-loop sequences

| Grouping | *n* | *N* | *Hd* | *π* | Tajima's D | Fu’s | SSD | Raggedness |
| --- | --- | --- | --- | --- | --- | --- | --- | --- |
| BL | 25 | 2 | 0.08 | 0.0053 | -1.51406* | -0.22919^ns^ | 0.00916^ns^ | 0.8592^ns^ |
| AQ | 27 | 2 | 0.074 | 0.0025 | -1.15354^ns^ | -1.12456** | 0.00002^ns^ | 0.73114^ns^ |
| QL | 26 | 2 | 0.28 | 0.0096 | -0.95988^ns^ | -1.04649^ns^ | 0.00686^ns^ | 0.2703^ns^ |
| BX | 31 | 3 | 0.288 | 0.0120 | -1.20989^ns^ | -0.53765^ns^ | 0.0004^ns^ | 0.26876^ns^ |
| MD | 30 | 1 | 0 | ----- | 0 | 0 | 0 | 0 |
| BY | 34 | 9 | 0.775 | 0.0486 | -0.43214^ns^ | -2.62112^ns^ | 0.1104* | 0.0614^ns^ |
| EH | 34 | 11 | 0.832 | 0.0677 | 0.43793 ^ns^ | -3.75535* | 0.00152^ns^ | 0.01941^ns^ |
| WL | 39 | 4 | 0.499 | 0.0334 | -0.39231^ns^ | 0.65587 ^ns^ | 0.38425** | 0.35288^ns^ |
| HW | 8 | 2 | 0.429 | 0.0143 | 0 | 0.53626^ns^ | 0.01015^ns^ | 0.20408^ns^ |
| MM | 7 | 4 | 0.714 | 0.0381 | -1.43414 | -1.2166 ^ns^ | 0.00073^ns^ | 0.05896^ns^ |
| CG | 54 | 3 | 0.073 | 0.0049 | -1.8549** | -1.87582* | 0.00189^ns^ | 0.79652^ns^ |
| BT | 29 | 7 | 0.709 | 0.0516 | -0.39383^ns^ | -1.3166 ^ns^ | 0.12889* | 0.45184** |
| TG | 29 | 5 | 0.47 | 0.0229 | -0.83673^ns^ | -1.64848^ns^ | 0.00289^ns^ | 0.09913^ns^ |
| BO | 26 | 6 | 0.652 | 0.0536 | 0.88872 ^ns^ | -0.45865^ns^ | 0.01574^ns^ | 0.0599^ns^ |
| TM | 40 | 6 | 0.697 | 0.0347 | -0.69912^ns^ | -1.16657^ns^ | 0.00682^ns^ | 0.08482^ns^ |
| HL | 32 | 5 | 0.673 | 0.0575 | -0.78329^ns^ | 0.95704^ns^ | 0.05336^ns^ | 0.1882^ns^ |
| DR | 30 | 5 | 0.405 | 0.0338 | -0.94345^ns^ | -0.59415^ns^ | 0.24543^ns^ | 0.37138^ns^ |
| AL | 52 | 5 | 0.479 | 0.0293 | -1.06849 ^ns^ | -0.42884^ns^ | 0.00749^ns^ | 0.12039^ns^ |
| BE | 35 | 3 | 0.398 | 0.0256 | -0.46562^ns^ | 0.69513^ns^ | 0.26833** | 0.53725^ns^ |
| HH | 21 | 4 | 0.557 | 0.0269 | -1.0189 ^ns^ | -0.44008^ns^ | 0.00094^ns^ | 0.07363^ns^ |
| TS | 51 | 1 | 0 | ----- | 0 | 0 | 0 | 0 |
| BG | 56 | 5 | 0.321 | 0.0049 | -1.1163 ^ns^ | -2.09323^ns^ | 0.00417^ns^ | 0.25922^ns^ |
| SH | 30 | 2 | 0.125 | 0.0042 | -0.77374 ^ns^ | -0.46749^ns^ | 0.04243^ns^ | 0.57886^ns^ |
| overall | 746 | 30 | 0.728 | 0.0021 | -1.5135* | -18.5425** | 0.013** | 0.0531^ns^ |

*n*, sample size; *N*, number of haplotypes; *Hd*, haplotype diversity; *π*, nucleotide diversity; Tajima’s D, Tajima’s D value; Fu’s, Fu and Li’s D value; SSD, goodness-of-fit to a simulated population expansion; and Raggedness, Harpending’s Raggedness index estimated under demographic expansion model. (ns: P > 0.05, *: P < 0.05, **: P < 0.01)

**Table S7** Pairwise *F_ST_* values calculated based on microsatellite data between 23 geographic locations sampled

|  | BL | AQ | QL | BX | MD | BY | EH | WL | HW | MM | CG | BT | TG | BO | TM | HL | DR | AL | BE | HH | TS | BG | SH |
| --- | --- | --- | --- | --- | --- | --- | --- | --- | --- | --- | --- | --- | --- | --- | --- | --- | --- | --- | --- | --- | --- | --- | --- |
| BL | 0.000 |  |  |  |  |  |  |  |  |  |  |  |  |  |  |  |  |  |  |  |  |  |  |
| AQ | 0.087 | 0.000 |  |  |  |  |  |  |  |  |  |  |  |  |  |  |  |  |  |  |  |  |  |
| QL | 0.100 | 0.056 | 0.000 |  |  |  |  |  |  |  |  |  |  |  |  |  |  |  |  |  |  |  |  |
| BX | 0.074 | 0.037 | 0.021 | 0.000 |  |  |  |  |  |  |  |  |  |  |  |  |  |  |  |  |  |  |  |
| MD | 0.095 | 0.087 | 0.070 | 0.053 | 0.000 |  |  |  |  |  |  |  |  |  |  |  |  |  |  |  |  |  |  |
| BY | 0.079 | 0.053 | 0.025 | 0.015 | 0.065 | 0.000 |  |  |  |  |  |  |  |  |  |  |  |  |  |  |  |  |  |
| EH | 0.070 | 0.040 | 0.032 | 0.018 | 0.053 | 0.022 | 0.000 |  |  |  |  |  |  |  |  |  |  |  |  |  |  |  |  |
| WL | 0.067 | 0.043 | 0.024 | 0.015 | 0.066 | 0.020 | 0.021 | 0.000 |  |  |  |  |  |  |  |  |  |  |  |  |  |  |  |
| HW | 0.119 | 0.107 | 0.058 | 0.048 | 0.085 | 0.042 | 0.070 | 0.063 | 0.000 |  |  |  |  |  |  |  |  |  |  |  |  |  |  |
| MM | 0.088 | 0.051 | 0.044 | 0.031 | 0.062 | 0.041 | 0.040 | 0.042 | 0.069 | 0.000 |  |  |  |  |  |  |  |  |  |  |  |  |  |
| CG | 0.086 | 0.069 | 0.065 | 0.051 | 0.072 | 0.045 | 0.047 | 0.059 | 0.072 | 0.059 | 0.000 |  |  |  |  |  |  |  |  |  |  |  |  |
| BT | 0.070 | 0.050 | 0.034 | 0.020 | 0.058 | 0.022 | 0.028 | 0.024 | 0.057 | 0.038 | 0.046 | 0.000 |  |  |  |  |  |  |  |  |  |  |  |
| TG | 0.111 | 0.048 | 0.065 | 0.038 | 0.070 | 0.043 | 0.039 | 0.054 | 0.099 | 0.054 | 0.059 | 0.042 | 0.000 |  |  |  |  |  |  |  |  |  |  |
| BO | 0.069 | 0.046 | 0.043 | 0.017 | 0.053 | 0.034 | 0.029 | 0.032 | 0.064 | 0.039 | 0.060 | 0.032 | 0.046 | 0.000 |  |  |  |  |  |  |  |  |  |
| TM | 0.068 | 0.052 | 0.038 | 0.023 | 0.056 | 0.028 | 0.026 | 0.031 | 0.072 | 0.035 | 0.040 | 0.028 | 0.043 | 0.026 | 0.000 |  |  |  |  |  |  |  |  |
| HL | 0.078 | 0.063 | 0.055 | 0.037 | 0.075 | 0.033 | 0.042 | 0.050 | 0.063 | 0.048 | 0.035 | 0.028 | 0.047 | 0.039 | 0.029 | 0.000 |  |  |  |  |  |  |  |
| DR | 0.092 | 0.066 | 0.054 | 0.036 | 0.071 | 0.044 | 0.039 | 0.052 | 0.081 | 0.044 | 0.058 | 0.041 | 0.054 | 0.032 | 0.025 | 0.043 | 0.000 |  |  |  |  |  |  |
| AL | 0.074 | 0.049 | 0.047 | 0.031 | 0.064 | 0.038 | 0.032 | 0.040 | 0.082 | 0.040 | 0.052 | 0.035 | 0.047 | 0.030 | 0.020 | 0.034 | 0.030 | 0.000 |  |  |  |  |  |
| BE | 0.080 | 0.057 | 0.067 | 0.042 | 0.087 | 0.056 | 0.048 | 0.055 | 0.101 | 0.057 | 0.080 | 0.043 | 0.061 | 0.033 | 0.037 | 0.053 | 0.041 | 0.027 | 0.000 |  |  |  |  |
| HH | 0.085 | 0.055 | 0.056 | 0.039 | 0.072 | 0.051 | 0.041 | 0.049 | 0.094 | 0.045 | 0.067 | 0.046 | 0.062 | 0.037 | 0.038 | 0.056 | 0.043 | 0.023 | 0.030 | 0.000 |  |  |  |
| TS | 0.069 | 0.072 | 0.081 | 0.052 | 0.105 | 0.074 | 0.059 | 0.052 | 0.121 | 0.069 | 0.111 | 0.067 | 0.095 | 0.057 | 0.066 | 0.087 | 0.082 | 0.058 | 0.051 | 0.051 | 0.000 |  |  |
| BG | 0.096 | 0.054 | 0.065 | 0.047 | 0.097 | 0.067 | 0.048 | 0.056 | 0.116 | 0.059 | 0.104 | 0.065 | 0.074 | 0.047 | 0.055 | 0.080 | 0.064 | 0.034 | 0.039 | 0.030 | 0.044 | 0.000 |  |
| SH | 0.143 | 0.124 | 0.121 | 0.100 | 0.121 | 0.115 | 0.092 | 0.115 | 0.153 | 0.085 | 0.121 | 0.093 | 0.091 | 0.100 | 0.092 | 0.092 | 0.089 | 0.076 | 0.076 | 0.071 | 0.099 | 0.081 | 0.000 |

**Table S8** Clades support and ancestral areas reconstruction as obtained with S-DIVA and Lagrange

|  | **S-DIVA** | | | **Lagrange** | | |
| --- | --- | --- | --- | --- | --- | --- |
| Node | area | Relative  probablity [%] | P | Split | Relative probablity | InL |
| 37 | SE | 100 | 0.19 | [SE\|SE] | 1 | -57.24 |
| 38 | SE | 100 | 0.63 | [SE\|SE] | 0.518 | -57.9 |
|  |  |  |  | [SE\|W,NE,SE] | 0.141 | -59.2 |
|  |  |  |  | [SE\|NE,SE] | 0.127 | -59.3 |
| 39 | SE | 100 | 0.07 | [SE\|SE] | 0.704 | -57.6 |
|  |  |  |  | [NE,SE\|SE] | 0.256 | -58.6 |
| 40 | SE | 100 | 0 | [SE\|SE] | 0.8 | -57.46 |
| 41 | SE | 100 | 0.14 | [SE\|SE] | 0.395 | -58.17 |
|  |  |  |  | [W,NE,SE\|SE] | 0.228 | -58.72 |
| 42 | SE | 100 | 0.03 | [SE\|SE] | 1 | -57.24 |
| 43 | SE | 100 | 0.01 | [SE\|SE] | 0.8421 | -57.41 |
| 44 | W,SE | 100 | 0.08 | [W\|SE] | 1 | -57.24 |
| 45 | SE | 100 | 0 | [SE\|SE] | 0.97 | -57.27 |
| 46 | SE | 100 | 0.05 | [SE\|SE] | 1 | -57.24 |
| 47 | SE | 100 | 0 | [SE\|SE] | 0.986 | -57.26 |
| 48 | SE | 100 | 0.04 | [SE\|SE] | 0.917 | -57.33 |
| 49 | SE | 100 | 0.39 | [SE\|SE] | 0.663 | -57.65 |
|  |  |  |  | [NE,SE\|SE] | 0.298 | -58.45 |
| 50 | SE | 100 | 0.96 | [SE\|SE] | 1 | -57.24 |
| 51 | SE | 100 | 0.46 | [SE\|SE] | 0.79 | -57.48 |
|  |  |  |  | [NE,SE\|SE] | 0.186 | -58.92 |
| 52 | NE,SE | 100 | 0.54 | [SE\|NE] | 0.8 | -57.47 |
|  |  |  |  | [NE,SE\|NE] | 0.184 | -58.94 |
| 53 | SE | 92.86 | 0.28 | [SE\|SE] | 0.66 | -57.66 |
|  | NE,SE | 5.36 |  | [SE\|NE,SE] | 0.193 | -58.88 |
|  | W,SE | 1.79 |  |  |  |  |
| 54 | W | 83.88 | 0.06 | [W,NE\|W] | 0.332 | -58.35 |
|  | W,NE | 16.67 |  | [W\|W] | 0.253 | -58.62 |
|  |  |  |  | [W,NE,SE\|W] | 0.25 | -58.63 |
| 55 | W | 70.83 | 0.04 | [W,NE\|W] | 0.449 | -58.04 |
|  | W,NE | 20.83 |  | [W\|W] | 0.319 | -58.38 |
|  | W,SE | 8.33 |  | [W,NE,SE\|W] | 0.141 | -59.2 |
| 56 | NE,SE | 100 | 0.02 | [NE\|NE] | 0.496 | -57.94 |
|  | W,NE | 0 |  | [W\|NE] | 0.342 | -58.31 |
| 57 | W | 55.47 | 1 | [W,NE,SE\|SE] | 0.106 | -59.49 |
|  | NE | 44.53 |  | [SE\|W,NE,SE] | 0.106 | -59.49 |
|  |  |  |  | [W,NE,SE\|NE] | 0.106 | -59.49 |
| 58 | W | 61.11 | 0.03 | [W\|W] | 0.293 | -58.47 |
|  | W,NE | 27.78 |  | [W,NE\|W] | 0.258 | -58.6 |
|  | W,SE | 11.11 |  | [W,NE,SE\|W] | 0.23 | -58.71 |
| 59 | W | 60 | 0.05 | [W,NE\|W] | 0.342 | -58.31 |
|  | W,NE | 40 |  | [NE\|NE] | 0.266 | -58.56 |
| 60 | W,NE | 100 | 0.02 | [W,NE\|NE] | 0.492 | -57.95 |
|  |  |  |  | [NE\|NE] | 0.325 | -58.37 |
|  |  |  |  | [W,NE,SE\|NE] | 0.103 | -59.52 |
| 61 | SE | 67 | 1 | [SE\|W,SE] | 0.699 | -57.6 |
|  | W,SE | 33 |  | [SE\|SE] | 0.227 | -58.72 |
| 62 | W,NE | 56.9 | 0.79 | [W\|W] | 0.2 | -58.85 |
|  | NE,SE | 23.32 |  | [NE\|W,SE] | 0.156 | -59.1 |
|  | W,SE | 19.77 |  | [W,NE\|SE] | 0.146 | -59.17 |
| 63 | NE | 66.03 | 0.79 | [NE\|W,NE,SE] | 0.534 | -57.87 |
|  | NE,SE | 26.79 |  | [NE\|W,SE] | 0.136 | -59.23 |
|  |  |  |  | [NE\|W,NE] | 0.126 | -59.31 |
| 64 | NE | 62.03 | 0.54 | [W,NE,SE\|NE] | 0.135 | -59.24 |
|  | NE,SE | 12.82 |  |  |  |  |
| 65 | SE | 63.47 | 1 | [SE\|W,NE,SE] | 0.41 | -58.15 |
|  | NE,SE | 20.9 |  | [SE\|W,NE] | 0.35 | -58.29 |
|  | W,SE | 8.67 |  | [SE,NE \|W] | 0.12 | -59.29 |

Node numbers refer to Fig. 3D. The column of Split represents the estimated ancestral areas for the branches following the respective node. Vertical bars separate upper branches (left) from lower branches (right). Only estimates within one log-likelihood unit of the optimal value are given. The relative probabilities (< 0.1) were disregarded. W, NE and SE are the abbreviated form of West, Northeast and Southeast (Fig. 3C).
