# Supplementary figures and images for: The colonization and divergence patterns of Brandt’s vole (Lasiopodomys brandtii) populations reveal evidence of genetic surfing
Source: BMC Evol Biol. 2017 Jun 21;17:145. doi: 10.1186/s12862-017-0995-y (PMC5480173; doi:10.1186/s12862-017-0995-y)

**A**

Mean of est. Ln prob of data

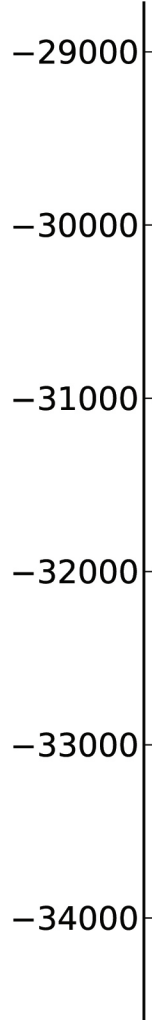**B**

Delta K

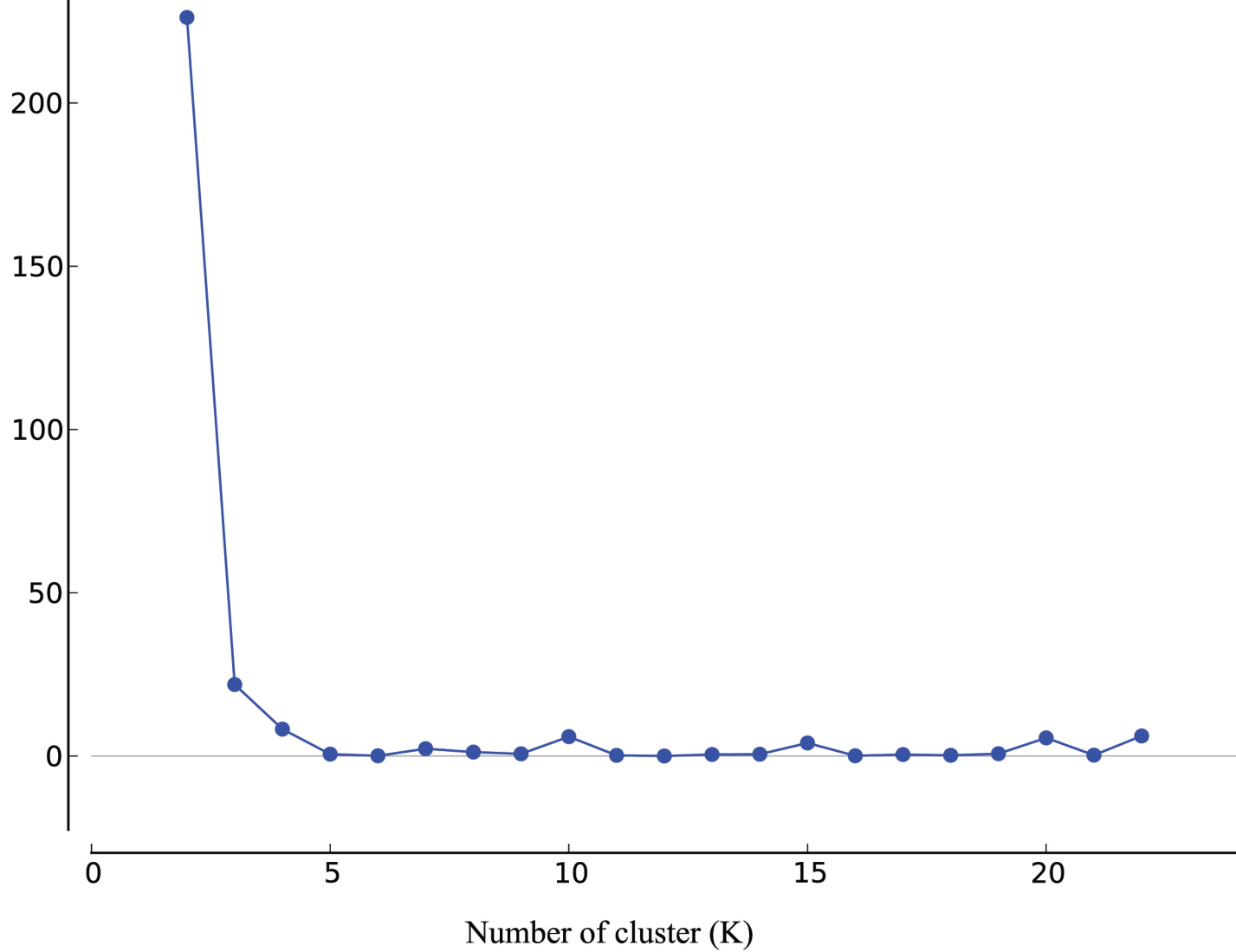

Supplement: Supplementary file 2 — Brandt’s vole population structure based on 12 microsatellites loci as implemented by STRUCTURE. A) Values of lnP(D) from 20 independent runs for K = 1–23. B) Values of ΔK from 20 independent runs plotted for K = 1–23 and calculated by the Evanno method. (PDF 3121 kb) [file 12862_2017_995_MOESM2_ESM.pdf]
